# The Emerging Role of p21 in Diabetes and Related Metabolic Disorders

**DOI:** 10.3390/ijms252313209

**Published:** 2024-12-09

**Authors:** Omar Elmitwalli, Radwan Darwish, Lana Al-Jabery, Ahmed Algahiny, Sornali Roy, Alexandra E. Butler, Ammar S. Hasan

**Affiliations:** 1 Department of Medicine, Royal College of Surgeons in Ireland—Medical University of Bahrain Busaiteen, Adliya P.O. Box 15503, Bahrain; 18244271@rcsi-mub.com (O.E.); 22200493@rcsi-mub.com (R.D.); 20202463@rcsi-mub.com (L.A.-J.); 19201282@rcsi-mub.com (A.A.); 17220637@rcsi-mub.com (S.R.); 2 Department of Postgraduate Studies and Research, Royal College of Surgeons in Ireland—Medical University of Bahrain Busaiteen, Adliya P.O. Box 15503, Bahrain; ahasan@rcsi-mub.com

**Keywords:** p21, metabolism, diabetes, insulin resistance, therapeutic strategies

## Abstract

In the context of cell cycle inhibition, anti-proliferation, and the dysregulation observed in certain cancer pathologies, the protein p21 assumes a pivotal role. p21 links DNA damage responses to cellular processes such as apoptosis, senescence, and cell cycle arrest, primarily functioning as a regulator of the cell cycle. However, accumulating empirical evidence suggests that p21 is both directly and indirectly linked to a number of different metabolic processes. Intriguingly, recent investigations indicate that p21 significantly contributes to the pathogenesis of diabetes. In this review, we present a comprehensive evaluation of the scientific literature regarding the involvement of p21 in metabolic processes, diabetes etiology, pancreatic function, glucose homeostasis, and insulin resistance. Furthermore, we provide an encapsulated overview of therapies that target p21 to alleviate metabolic disorders. A deeper understanding of the complex interrelationship between p21 and diabetes holds promise for informing current and future therapeutic strategies to address this rapidly escalating health crisis.

## 1. Introduction

The worldwide prevalence of diabetes mellitus presents a major public health issue. A total of 536.6 million people worldwide (10.5%) had diabetes in 2021, according to the International Diabetes Federation (IDF) [1]. The number of patients with diabetes is expected to rise, with projections suggesting that 643 million will be afflicted by 2030 and 783 million by 2045 [1]. Type 2 diabetes mellitus (T2DM) represents approximately 96% of all diabetes cases worldwide [2]. Therefore, understanding the pathogenesis of T2DM is essential to finding novel approaches to prevent and/or treat the disease.

Insulin resistance is a hallmark of T2DM, primarily characterized by impaired insulin signaling. This dysfunction affects the insulin receptor substrate (IRS), phosphoinositide-3-kinase (PI-3K), and the protein kinase B (PKB) axis, reducing the effect of insulin on target tissue, primarily skeletal muscle, liver, and adipose tissue [3]. Obesity is the main promoter of insulin resistance, where adipose tissue releases greater quantities of non-esterified fatty acids, glycerol, hormones and pro-inflammatory cytokines. However, some individuals with obesity remain insulin sensitive [4,5].

Further, the tumor suppressor protein p53 activates the DNA damage-induced checkpoint by transactivating genes that enforce cell cycle arrest [6]. Of particular interest, the p21WAF1/Cip1 protein (p21), encoded by the cyclin-dependent kinase inhibitor 1A (*CDKN1A*) gene, mediates p53-dependent G1 growth arrest [7]. p21 carries out its biological functions by binding to and inhibiting cyclin-dependent kinases (CDKs) within the cell. It also inhibits Proliferating Cell Nuclear Antigen (PCNA)-dependent DNA polymerase activity by binding to PCNA, preventing DNA replication and modulating a number of PCNA-dependent DNA repair mechanisms [8].

p21 is also involved in metabolic processes that include cellular senescence, DNA damage repair, response to cellular stress, and the regulation of apoptosis [9,10,11,12]. To do this, p21 also interacts with p53, the Signal Transducer and Activator of Transcription 3 (STAT3), c-Myc oncoprotein (c-Myc), and E2F Transcription Factor 1 (E2F1), among others [13,14,15,16]. Its involvement in the adenosine monophosphate (AMP)-activated protein kinase (AMPK) pathway hints at a link between stress responses and metabolic adaptation. Moreover, p21 is connected to the rat sarcoma (RAS)/rapidly accelerated fibrosarcoma (RAF)/mitogen-activated protein kinase kinase (MEK)/extracellular-signal regulated kinase (ERK) pathway, which impacts cell proliferation and survival, suggesting a role in cell growth and metabolic decision-making [17,18,19].

Targeting p21 for suppression emerges as a promising strategy to preserve functional β-cell mass, seeing that p21 inhibits β-cell proliferation, triggers apoptosis, and degrades β-cell mass. Given its significant role in diabetes and metabolic disorders, finding ways to modulate p21 could lead to new therapeutic approaches that slow or prevent the loss of β-cell mass and function. This review thoroughly examines p21’s involvement in metabolism, with a particular emphasis on its role in diabetes.

## 2. Structure and Function of p21, the Cell Cycle Inhibitor

### 2.1. p21 Structure and Protein Network

The human p21 protein consists of 164 amino acids and its gene is located on chromosome 6 in the 6p21.2 region in the human cell. p21 is an intracellular protein expressed in all mammalian cells, but its levels and activity vary depending on the context. p21 expression is largely dependent on ongoing cellular processes and it plays a major role in stress responses; therefore, p21 expression is generally higher in tissues that experience frequent stress, rapid turnover, or require tight regulation of the cell cycle for differentiation or repair processes. The transcription of p21 can be p53-dependent or p53-independent and is induced in response to stimuli; cell stress, DNA damage, and chemicals are all examples of stimuli that promote the p53-dependent upregulation of p21. Furthermore, p21 can interact with several proteins involved in an array of important biological processes within the cell because it lacks a clearly defined tertiary structure [20,21,22]. p21 is regulated by transcriptional factors [23,24] and post-transcriptional regulators such as microRNAs (miRNAs) and RNA binding proteins [25], and undergoes post-translational modifications [26]. Apart from its role as a cell cycle regulator, p21 regulates DNA replication, repair, and apoptosis [21], and inhibits tumor growth as a tumor suppressor gene [27]. The presence or absence of p21 is largely dependent on the cellular processes taking place, but it is not ubiquitous across all cell types. However, it is not specific to certain cell types either; instead, it plays a role in stress responses [28].

Several domains within p21 help facilitate these functions. First, the PCNA binding domain enables p21 to regulate DNA replication and repair processes by inhibiting DNA synthesis, suppressing mutagenesis and promoting DNA repair [8]. p21 also contains two distinct cyclin-binding motifs, Cy1 and Cy2, which independently interact with cyclins to inhibit cyclin-Cdk kinases and suppress cell growth [29,30,31]. The nuclear localization sequence (NLS) guides p21 to the cell nucleus, where it regulates the cell cycle and participates in DNA damage responses [32]. Furthermore, p21 inhibits the transcriptional activity of c-Myc via its c-Myc binding domain, suppressing cell proliferation [29,33]. Calmodulin binding domains regulate p21 intracellular localization and phosphorylation [29,34]. Collectively, these domains contribute to the diverse functions and regulatory roles of p21.

**Figure 1:**
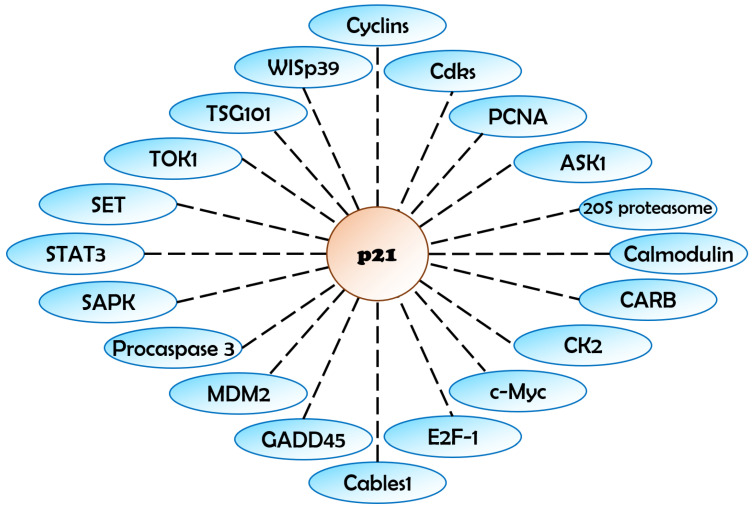
Network of protein interactions involving p21 in diverse cellular processes. Protein interaction network of p21. The dotted line describes reported protein interactions, whether that interaction was direct or indirect. WISp39: WISP3 (WNT1 Inducible Signaling Pathway Protein 3), TSG101: TSG101 (Tumor Susceptibility Gene 101), TOK1: TOK1 (K+ Transporter TOK1), SET: SET (SET Nuclear Proto-Oncogene), STAT3: STAT3 (Signal Transducer and Activator of Transcription 3), SAPK: MAPK8 (Mitogen-Activated Protein Kinase 8), Procaspase3: CASP3 (Caspase 3), MDM2: MDM2 (MDM2 Proto-Oncogene), GADD45: GADD45A (Growth Arrest and DNA Damage-Inducible Alpha), Cables1: CABLES1 (Cdk5 and Abl Enzyme Substrate 1), E2F-1: E2F1 (E2F Transcription Factor 1), c-Myc: MYC (MYC Proto-Oncogene), CK2: CSNK2A1 (Casein Kinase 2 Alpha 1), CARB: N/A (context needed), Calmodulin: CALM1, CALM2, CALM3 (Calmodulin 1, 2, 3), 20S proteasome: PSMB5 (Proteasome Subunit Beta 5), ASK1: MAP3K5 (Mitogen-Activated Protein Kinase Kinase 5), PCNA: PCNA (Proliferating Cell Nuclear Antigen).

The functional diversity of p21 is mediated through its complex network of protein interactions [35], captured in Figure 1. Different regions of p21 are responsible for binding to different proteins, as detailed in Table 1. For instance, the N-terminal domain of p21 inhibits cyclin-Cdk kinases, while the C-terminal domain inhibits PCNA to modulate DNA replication and cell growth, particularly under the conditions of cellular stress [36,37]. Additionally, the hydroxyl group of Tyr151 within p21 serves as a tethering point, facilitating precise recognition and alignment at the peptide–protein interface, and optimizing its binding affinity for PCNA [37]. Further, the C-terminus of p21 contains motifs that are crucial for regulating the function of the p300 sumoylation-dependent transcriptional repression domain, CRD1 [36]. Understanding the specific interactions and their implications provides valuable insight into the multifaceted role of p21 in cellular physiology.

### 2.2. p21 Protein Regulation

The primary transcriptional regulator of p21 is p53. Two conserved p53 responsive elements (p53RE) are found in the promoter of p21 [63]. p21 expression is activated by various molecules and transcription factors independently of p53. These factors interact with specific elements in the p21 promoter region when stimulated by signals like butyrate, phorbol myristate acetate (PMA), and the nerve growth factor (NGF) [24]. For instance, the retinoblastoma protein (pRB) [64], Sp1, and Sp3 [65], as well as double homeobox 4 (Dux4) and caudal-type homeobox 2 (CDX2), a member of the caudal-related homeobox gene family, enhance p21 expression by activating its promoter region [66,67]. In a p53-independent manner, Cdk5 and Abl Enzyme Substrate 1(Cables1) inhibit proteasomal degradation, promoting the stabilization of p21 in conjunction with other cell cycle regulators [62]. Furthermore, integrin β1 enhances p21 transcription by recruiting Sp1 to the p21 promoter region [68]. Additionally, Kruppel-like zinc finger transcription factor 6 (Klf6), a member of the Klf family, is recruited to the p21 promoter region and is acetylated by p300-CREBBP to initiate p21 transcription [69].

As for transcription factors, these include the signal transducers and activators of transcription (STAT), E2F-1/E2F-3, Smads, AP2, BETA2, GAX, CCAAT/enhancer binding protein-α (C/EBPα), C/EBPβ, and myoblast determination protein 1 (MYOD1) [24]. Extracellular antiproliferative signals such as transforming growth factor-β (TGF-β) can also activate transcription factors, including Sp1 and Smads, to induce p21 expression independently of p53 [70].

Conversely, p21 inhibits cyclin-dependent kinase activities directly by interacting with their N-terminal domains or indirectly by interfering with CDK1 and CDK2 phosphorylation [65]. The N-terminal p21 region has a crucial cyclin-binding motif 1 (Cy1) for cyclin subunit interaction and a weaker redundant motif, Cy2, in the C-terminal region, both pivotal for inhibiting cyclin-CDK complexes [71]. Furthermore, p21 downregulates MYC and CDC25A genes by binding to their promoters, recruited by STAT3 and E2F in the DNA damage response. This recruitment reduces histone H4 acetylation and inhibits p300 histone acetylase recruitment [72]. Moreover, p21 regulates several genes associated with cell division, senescence, and aging, including t-tGase, cathepsin B, PAI-1, fibronectin, and N-acetylgalactosamine-6-sulfate sulfatase [73]. Additionally, as part of the p53-DREAM pathway, p21 indirectly downregulates the genes involved in DNA repair, apoptosis and cell cycle regulation, such as BRCA1, BRCA2, H2AX, NET1, STK17B, E2F1, CHEK2 and CDK1 [74,75]. All p21 protein interactions are captured in Table 1 above.

## 3. Diabetes and Metabolic Disorders

Diabetes is a chronic metabolic condition characterized by elevated levels of glucose in the blood. There are several types of diabetes, the most common being type 1 diabetes mellitus (T1DM), T2DM, and gestational diabetes mellitus (GDM) [76]. T1DM is precipitated by the autoimmune destruction of the β-cells in the pancreas while T2DM is due to the mismatch between insulin levels and insulin sensitivity, impairing the body’s response to insulin and progressively reducing β-cell mass and function. T1DM is typically diagnosed in childhood and T2DM in adulthood based on two abnormal glucose readings in the case of ambiguous symptoms. The criteria to diagnose diabetes are fasting plasma glucose ≥ 7.0 mmol/L, or 2 h PG ≥ 11.1 mmol/L during the oral glucose tolerance test (OGTT), or HbA1C ≥ 6.5% (48 mmol/mol) [76]. Given that T2DM accounts for approximately 96% of diabetes cases [2], eclipsing the less common forms [77], the focus here is primarily on this most prevalent form.

β-cell mass refers to the total amount of β-cells in the pancreas and a sufficient β-cell mass is crucial for appropriate insulin production and maintenance of glucose homeostasis [78]. In contrast, β-cell function refers to the ability of β-cells to release insulin in response to fluctuations in blood glucose levels. An adequate β-cell mass enhances insulin production capacity, while compromised β-cell function, resulting from inflammation, oxidative stress, endoplasmic reticulum (ER) stress, excess cytokine secretion, and genetic aberrations, can inhibit insulin production [79].

Any reduction in β-cell mass places a heightened burden on the remaining cells, potentially provoking β-cell dysfunction, reduced insulin production and, ultimately, T2DM. Preserving β-cell mass and function is crucial for T2DM management through lifestyle changes, and medications targeting insulin secretion and β-cell protection [80]. In T2DM, the loss of β-cells occurs by apoptosis, leading to inadequate insulin secretion and elevated glucose levels [81,82]. Current evidence suggests that p21 expression increases when β-cells are under stress, potentially contributing to apoptosis by activating pro-apoptotic pathways and inhibiting anti-apoptotic ones [83,84].

Cell stress is a broad term used to describe situations where a cell faces disruptions to its normal functioning or survival. These challenges can arise from environmental changes, chemotherapy, radiation, oxidative stress, and ER stress. In response to these stressors, cells activate various responses, including heat shock responses, the unfolded protein response (UPR), DNA damage, and oxidative stress responses, to adapt and maintain cellular homeostasis. Cellular damage or death results in the setting of prolonged or severe stress responses [85].

β-cell stress refers specifically to the stress experienced by insulin-secreting β-cells in the pancreas [86], and can be attributed to a number of different causes. First, glucose toxicity due to prolonged high blood glucose leads to reactive oxygen species (ROS) accumulation, inducing oxidative stress and damage [87]. Second, lipotoxicity, induced by elevated free fatty acids in the bloodstream causing lipid build-up impairs insulin secretion [88]. Third, chronic inflammation, prompting the release of inflammatory cytokines such as interleukin-1 beta (IL-1β) and tumor necrosis factor alpha (TNF-α), intensifies β-cell stress and induces apoptosis [89]. Fourth, disruptions in protein folding and assembly within the ER, causing ER stress and activating the UPR, induce β-cell dysfunction and, ultimately, death [86,90]. Fifth, mutations in vital genes encoding insulin, MafA, Pdx1 and FoxO1, crucial for β-cell function and survival, trigger stress and dysfunction [91,92,93]. All these factors collectively contribute to impaired insulin secretion and eventual β-cell death, characteristic features of T2DM [86,87,88,89,90,91].

## 4. The Cell Cycle Regulator p21 Influences Diabetes

Cell cycle progression is not random; it is checkpoint-regulated by cyclin-dependent kinases (CDKs). During the G1/S transition, mitogenic factors activate intracellular signaling networks involving D-type cyclins (D1, D2, D3) binding to CDK4/6 and forming the cyclin D/CDK4/6 complex. This complex phosphorylates the tumor suppressor protein RB, releasing E2F transcription factors that drive the cell into the S phase [94,95].

CDK4/6 is regulated by two families of inhibitory proteins: INK4 and Cip/Kip. INK4 (comprising p15, p16, p18, and p19) hinders the binding of cyclin D to CDK4/6. Meanwhile, the Cip/Kip family deactivates CDK-cyclin complexes, preventing them from phosphorylating RB1. Among the Cip/Kip family members, p21 inhibits progression into the S phase by binding to PCNA [96]. The C-terminal domain of p21 is sufficient to inhibit DNA synthesis by displacing the enzymes associated with PCNA; however, this was never demonstrated in vivo because p21 is degraded during the S phase and not at any earlier stage [97]. Additionally, as detailed earlier, p21 is induced by p53 and thus bears responsibility for the life or death of a cell [98].

p21’s established role in cell cycle arrest during the G1 phase was demonstrated by Brugarolas and colleagues, in which radiation was shown to enhance p21 activity [99]. p21 is crucial to the G2 phase, inhibiting CDK-activating kinases and maintaining cell arrest in the presence of DNA damage by controlling cyclin B1 degradation. CDK1 kinase activity depends on the phosphorylation of threonine 161, mediated by CDK-activating kinase. The degradation of cyclin B1, which normally activates CDK1 for mitosis, deactivates CDK1 [100]. Finally, p21 is essential in the formation of the cyclin D/CDK4 complex discussed earlier, facilitating its roles in phosphorylating RB1 and initiating the transition into the S phase [97].

In 1999, Kaneto and colleagues were the first to report the link between p21 and diabetes. They induced oxidative stress in isolated rat pancreatic islets with hydrogen peroxide which increased p21 mRNA expression and decreased insulin mRNA expression. In Zucker diabetic fatty rats, overexpressing p21 using adenovirus induced p21 mRNA expression and reduced insulin mRNA expression as diabetes developed [101].

Further, Mihailidou et al. demonstrated that the transcription factor C/EBP homologous protein (CHOP), which induces ER stress-related apoptosis in diabetes, also regulates the expression of p21. p21 was shown to be inversely correlated with CHOP and it therefore inhibited ER stress-associated damage [102]. This finding builds upon a previous study showing that increased insulin demand in pancreatic β-cells triggers ER stress, leading to cell senescence and disrupting glucose homeostasis [103]. Thus, the authors concluded that p21 can be utilized to adjust cell sensitivity to ER stress, potentially reducing senescence and apoptosis and improving outcomes in diabetes [104].

## 5. p21 Regulates Diabetes Phenotypes

### 5.1. Cell Stress

p21 is activated under the conditions of chronic cellular stress, specifically prolonged elevated levels of blood glucose [105], oxidative stress [101] or an accumulation of the human islet amyloid polypeptide (IAPP) [106,107]. When activated, p21 halts cell cycle progression [108] and inhibits DNA replication [109] via mechanisms explained previously.

p21 is also responsible for mediating autophagy in the setting of cell stress. Autophagy is a ubiquitous process that serves as a recycling mechanism to engulf cellular contents, including organelles, in membrane-bound vacuoles (autophagosomes) and deliver them to lysosomes for degradation via proteolytic enzymes within the lysosomal compartment [110]. Autophagy plays a crucial role in promoting cell survival by eliminating damaged organelles and protein aggregates, as well as maintaining bioenergetic homeostasis [111].

This can be observed during prolonged nutrient depletion, where anabolic activity is reduced [112]. In cardiac cell stress, p21 promotes protective autophagy, while its suppression worsens oxidative stress, inflammation and mitochondrial damage [113]. Further, p21 demonstrates a variable regulatory role in apoptosis based on the type of cell stressor and the nature of the tissue [65,112]. Inhibition of apoptosis by p21 has been noted in hepatocyte cell lines [107], as well as breast cancer cell lines [108] where apoptosis is stimulated by p53 overexpression [65]. In oxidative stress, p21 has a pro-survival role, and its loss increases p53-dependent and PUMA-mediated cell death [114]. In β-cells exposed to chronic hyperglycemia, elevated p21 levels are linked to increased apoptosis and caspase 3 cleavage, a key apoptotic marker [107]. Taken together, it is apparent that p21 has a variety of roles in cell stress; however, the effect is consistently anti-proliferative and pro-apoptotic in β-cell stress, leading to a decline in β-cell mass [105,107]. p21-mediated apoptosis in β-cells is initiated through the intrinsic apoptotic pathway and facilitated by the pro-apoptotic BAX and BAK proteins. The suppression of either or both proteins decreases p21-induced caspase 3 cleavage [107]. Moreover, the increase in p21 leads to a reduction in glucose-simulated insulin secretion and β-cell function due to cell senescence [107,115,116].

Additionally, ER stress occurs when misfolded proteins accumulate in the ER due to disruption in its function [117,118]. Conditions such as nutrient deficiency, hypoxia, hyperglycemia, and hypocalcemia raise protein synthesis demands, exceeding the ER’s folding capacity, thereby leading to ER stress. This activates the unfolded protein response (UPR) to restore ER balance by increasing chaperone production and reducing mRNA translation. Prolonged ER stress can cause the UPR to become pro-apoptotic, resulting in cell death [118,119].

ER stress and the pathways it triggers play a causal role in the pathogenesis of several diseases, including cancer, neurodegenerative disorders, ophthalmological diseases, and metabolic disorders [117]. In diabetes, the upsurge of insulin demand induces ER stress in the secretory pancreatic β-cells. As the stressors remain, and the ER stress becomes prolonged, the β-cells are ultimately destroyed via apoptosis, diminishing pancreatic endocrine function and compromising blood glucose homeostasis [104]. p21 plays a role in both the pro-survival and pro-apoptotic pathways in ER-stressed β-cells as p21 is expressed in conditions of moderate ER stress but suppressed in conditions of intense ER stress [104]. When p21 is upregulated, it exhibits pro-survival features by inhibiting ER stress-mediated tissue damage. When downregulated via CHOP, a transcription factor induced in sustained UPR, it lowers the apoptotic threshold of cells, exacerbating ER stress-mediated tissue damage and apoptosis [102,120,121]. It is worth noting that p21 upregulates the pro-apoptotic protein BAX as part of its induction of apoptosis [97,107]. Paradoxically, suppressing p21 leads to a greater frequency of apoptosis versus when p21 is induced [102,120]. p21’s role is highly nuanced, and influenced by different upstream regulatory pathways, but ultimately culminates in controlling stress responses. Indeed, there is a delicate balance between protecting cells in stress conditions and eliminating them when their viability is compromised.

### 5.2. Cell Senescence

Cell senescence can occur due to several extrinsic and intrinsic stimuli, and two key pathways regulate it: p16/pRb and p53/p21 [97]. Telomere shortening leads to gradual senescence, while DNA damage accelerates it. Other stressors inducing senescence include RAS activation, oxidative stress, radiation, nutrient depletion, toxins, mitochondrial malfunction, inflammation, and tissue damage [122,123,124]. Furthermore, some senescent cells demonstrate increased senescence-associated β-galactosidase (SA-β-Gal) activity and secrete pro-inflammatory factors that form a senescence-associated secretory phenotype (SASP) [116], causing chronic inflammation. This promotes the development of several age-related diseases, such as atherosclerosis, lung disease, and diabetes [123,124]. Moreover, senescence impairs tissue repair, with the accumulation of non-proliferative cells, leading to the aging and dysfunction of the tissue [123]. In T2DM, senescent cells play a dual role, acting as both a causative factor, and a consequence of the disease. Senescent cells disrupt pancreatic β-cell function, triggering tissue damage and inflammation through the SASP. Simultaneously, T2DM-induced metabolic changes like elevated glucose promote cellular senescence, creating a diabetes-related feed forward cycle [115,116].

Cell senescence is typically associated with disease but, in embryonic development, it serves a programmed role in limb growth and patterning, without any associated pathology [125]. In transgenic mice that lack p21, the dysfunction of the apical ectodermal ridge (AER) has been noted, and alterations in pattern formation and cellular senescence have been found, suggesting that p21 mediates and is integral to senescence in embryonic development [125].

Given that p21 inhibits cell cycle progression, it serves as one of the primary regulators of senescence. Its expression is upregulated through both p53-dependent and p53-independent mechanisms in response to various senescence-inducing stimuli that include toxin exposure [123]. Additionally, increased oxidative stress and telomere shortening are found in the adipose tissue of obese individuals, also leading to senescence [116].

In mice, fat senescence usually occurs in the white adipose tissue of obese models and results in increased insulin resistance and diabetes. p21 was observed to accumulate in senescent fat cells, and the ablation of p21 in adipose tissue improved insulin sensitivity and glucose tolerance while suppressing the SASP phenotypes. This indicates that the p53/p21 pathway is directly involved in insulin resistance [124]. Metabolic stress, due to insulin resistance and an increase in blood glucose, promotes p21 expression, accelerating β-cell senescence and causing an age-independent decline in function. Senescent β-cells displayed an increased expression of senescence markers (p21, p16), SASP markers (Ccl2, Cxcl2, IL1α, IL6, TNFα), and the two genes catalase and LDHA, typically inhibited in non-senescent β-cells. On the other hand, senescent β-cells demonstrated the down-regulation of β-cell identity genes (Ins1, Mafa, Neurod1, Nkx6.1, Pdx1), involved in cellular depolarisation and glycolysis, components of incretin signal pathways, and constituents of insulin granules (Ins1, Ins2, Slc30a8). These changes can be linked to the loss of β-cell function associated with senescence [116,124].

### 5.3. Amino Acid Deficiency

Amino acids are the building blocks of proteins that serve as enzymes, structural compounds, signaling mediators, and energy metabolites. Though the human body can synthesize several amino acids endogenously, referred to as non-essential amino acids, there are nine amino acids (histidine, isoleucine, leucine, lysine, methionine, phenylalanine, threonine, tryptophan, valine) that the body cannot synthesize and can only absorb from food, referred to as essential amino acids [126]. Inadequate protein intake and protein deficiencies resulting from metabolic disorders can lead to amino acid deficiency, which is observed to play a role in the pathophysiology of T2DM, among other diseases. Arginine, glutamine, leucine, and phenylalanine directly promote insulin secretion, with arginine also being linked to increased insulin sensitivity [127]. Amino acid deficiencies have been shown to upregulate the expression of specific proteins. Specifically, in the HepG2 cells deprived of the essential amino acid histidine, there is an increase in the expression of both p21 and p27. The integrated stress response kinase GCN2, which upregulates p21, is also activated, halting cell cycle progression and inhibiting cell proliferation [112,128]. In MCF10A mammary epithelial cells, withdrawing methionine resulted in an increase in the p21 levels, causing the cell to become quiescent. By comparison, the withdrawal of leucine or lysine resulted only in a moderate increase in the p21 levels. Nevertheless, even with the moderately increased levels of p21, leucine deficiency arrested the cell cycle, contrary to lysine [129].

### 5.4. Involvement in Obesity

Obesity typically results from excessive calorie intake and inadequate physical activity, although genetics, underlying medical conditions, and specific medications can also contribute to its development [130]. Adipocyte hyperplasia or hypertrophy, which leads to the increased storage of lipids in adipose tissue, is an important characteristic of obesity [131]. Obesity typically precedes the onset of insulin resistance which, in turn, can accelerate the onset of cardiovascular diseases and T2DM [130]. p21 has a role in promoting adipose tissue expansion in obesity and exacerbating insulin resistance by participating in adipocyte differentiation and hypertrophy [131]. p21 plays a role in adipocyte differentiation from fibroblasts, and its absence inhibits the later stage of adipocyte differentiation. Additionally, p21 prevents the apoptosis of hypertrophic adipocytes, and when absent, adipose tissue expansion is suppressed, and obesity is ameliorated [131].

In obese mice, p21 is overexpressed in gonadal visceral adipose tissue (gVAT) cell lines. While an initial increase in p21^high^ cells is observed in gVAT cell lines, liver hepatocytes subsequently exhibit elevated p21 levels, with no corresponding accumulation in muscle, brown fat, or the pancreas [94]. Moreover, obese mice display reduced insulin sensitivity and impaired glucose homeostasis. Notably, the elimination of p21^high^ cells significantly improved metabolic function, even in the advanced stages. The results were mirrored in humans with obesity, where the elimination of the p21^high^ cells in the visceral adipose tissue (VAT) improved the metabolic function. This suggests that p21 can be targeted therapeutically to reduce obesity-induced insulin resistance [94].

## 6. The Role of p21 in Glucose Homeostasis

### 6.1. p21’s Involvement in Pancreatic Function

p21 expression is vital for proper pancreatic function. Specifically, p21 levels increase in pancreatic islet cells during the onset of diabetes. The rise in p21 expression is likely to contribute to β-cell glucose toxicity by inhibiting both cell proliferation and insulin biosynthesis [101].

The transcription factor KLF10 is also involved in the regulation of pancreatic function via the SEI-1p21Cip1 pathway. Specifically, KLF10 binds to the p21 promoter and upregulates its expression which, in turn, regulates pancreatic β-cell function and survival [132]. Additionally, studies have revealed that the deficiency of KLF10 in mice leads to a reduction in pancreatic islet mass. Moreover, KLF10 has been identified as a regulator of acinar cell differentiation and proliferation via the SEI-1p21Cip1 pathway [132].

Research has revealed that p21’s response to DNA damage, induced by stress and metabolic disturbances, triggers cellular senescence and diminishes β-cell proliferation, ultimately contributing to the decline of pancreatic islet mass [133]. Glucose intolerance and hypoinsulinemia are exacerbated as p21 expression increases, leading to diabetes progression [86]. Stress-activated p21 has been identified as a factor responsible for β-cell mass reduction by stimulating intrinsic apoptotic pathways [86]. Surprisingly, p21’s overexpression can have both detrimental and beneficial effects on β-cells, as it exacerbates glucotoxicity-induced apoptosis while also promoting β-cell recovery after treatment with streptozotocin [104,134].

There is a paucity of studies that validate these findings in β-cells from human donors. However, one study that used non-diabetic human islets and the rat insulinoma INS 832/13 beta cell line observed that the deficiency of the p21 (Cdc42/Rac)-activated kinase (PAK1) induced glycemic dysregulation [135].

p21’s involvement in pancreatic function extends beyond metabolic disorders to pancreatic cancers. As pancreatic cancer transitions from normal tissue to adenocarcinoma, there is a significant upregulation of p21 expression in the carcinoma cells compared to the normal cells. This is met with an increase in p53. Together, p21 and p53 are crucial for regulating the cell cycle and inducing apoptosis in response to DNA damage. Their interaction plays a pivotal role in maintaining the integrity of pancreatic cells [136].

### 6.2. p21 Regulates Insulin Resistance

Obesity, often accompanied by chronic low-grade inflammation, is closely intertwined with insulin resistance and the onset of T2DM. Insulin resistance and hyperinsulinemia can also accelerate the onset of obesity [4]. One study showed that T2DM patients also experience an elevation in p21 levels, which, as detailed previously, correlates with impaired glucose metabolism. The removal of p21^high^ cells from adipose tissue successfully alleviates insulin resistance in obese mice [94].

While p21 plays a crucial role in the final stages of adipocyte differentiation and hypertrophy, adipogenesis can occur in its absence. p21 knockdown in hypertrophic adipose tissue and fully differentiated 3T3-L1 adipocytes triggers p53 activation and subsequent apoptosis. This highlights p21’s role in protecting hypertrophied adipocytes from apoptosis, allowing adipose tissue to expand, and exacerbating both obesity and insulin resistance [131]. Furthermore, p21 has been identified as an inhibitor of insulin signaling and glucose uptake in adipocytes, thus contributing to insulin resistance. It naturally follows that p21 deficiency in mice has been associated with protection against insulin resistance induced by a high-fat diet [131,137]. These findings warrant further mechanistic investigations into the relationship between p21 and insulin resistance.

### 6.3. p21 Regulates Glucose Tolerance

The role of p21 in glucose tolerance becomes apparent within the context of cellular senescence and its relevance to diabetes. In instances of high fat diet-induced diabetes accompanied by obesity, a distinct pattern emerges wherein adipose tissues experience an accumulation of p21^Cip1^-highly expressing p21^high^ cells during the early stage, followed by an increase in both p21^high^ and p16 ^Ink4a^-highly expressing (p16^high^) cells in the later stage. The removal of p21 ^high^ cells in visceral adipose tissue helps alleviate insulin resistance in obese mice [138]. That said, interventions targeting both p21^high^ and p16^high^ cells, such as the use of dasatinib plus quercetin, have improved glucose tolerance and reduced insulin resistance in immune-deficient mice transplanted with adipose tissue from obese patients [124,138].

Furthermore, the consideration of p21^Cip1^ as a viable candidate for senotherapy in obesity-linked diabetes stems from the fact that p21^high^ cells contribute to NF-kB dependent inflammation [94]. However, the implications of p21^high^ deletion on aging-related processes has been a subject of debate [139] partially attributed to the fact that, unlike p16^Ink4^, p21^Cip1^ plays a significant physiological role in vivo [140].

### 6.4. p21 Involvement in Glucose Starvation, Hyperglycemia, and Hypoglycemia

p21 mediates cellular adaptation to metabolic stress, especially in the cases of energy depletion caused by either starvation or the use of mitochondria respiration inhibitors. It has been reported that the effects of p21 activation in response to stress, such as cell cycle arrest and cell death, depend on the type, intensity, and duration of the specific stressor, as well as the type of cells affected [140]. Studies have revealed that short-term fasting increases the transcription of p21 in mice [141,142]. Additionally, Muñoz-Espín and colleagues demonstrated p21’s role in assisting stem cells in preserving their quiescent state and genomic integrity while preventing apoptosis. Nevertheless, under extended periods of stress, p21 can initiate apoptosis [140].

Under hyperglycemic conditions, insulin receptor substrate-1 (IRS-1) is downregulated in multiple cell types and insulin-like growth factor-I (IGF-I) signaling through IRS-1 is disrupted [143]. In the cell types that can undergo differentiation, such as vascular smooth muscle cells (VSMC), the downregulation of IRS-1 leads to the loss of P53/KLF4 association, which reduces the expression of myocardin and p21. This promotes VSMC differentiation and can accelerate atherosclerosis [144].

## 7. p21 Is Involved in Different Metabolic Pathways

### 7.1. p53/p21 Pathway and Warburg Effect

The distinct metabolic phenotype observed in cancer cells, known as the Warburg Effect, has long highlighted their metabolic divergence from normal cells. This phenomenon is characterized by increased glycolysis and decreased oxidative phosphorylation. In cancer cells, glucose uptake increases significantly, leading to persistent lactate production, despite the presence of oxygen and functional mitochondria. Extensively studied, the Warburg Effect underscores the unique energy utilization strategy of malignant cells [145].

Recent investigations have shed light on the role of p53 in regulating energy metabolism and the Warburg Effect. This function is predominantly carried out via the induction of *TP53*-induced glycolysis, the apoptosis regulator (TIGAR), and the Ras-related glycolysis inhibitor and calcium channel regulator (RRAD), which reduce glycolysis [146,147,148,149]. Additionally, p53 exerts transcriptional repression on GLUT1 and GLUT4, further reducing glycolysis [150]. Lastly, p53 plays a direct role in suppressing glucose metabolism by inhibiting glucose-6-phosphate dehydrogenase (G6PD), a key enzyme in the glycolysis pathway [151].

There is, however, a paucity of literature indicating the direct impact of p21 on the Warburg Effect’s pathways. Notably, Chu et al. also observed that the overexpression of miR-512-5p led to the knockdown of its target gene p21, inhibiting glycolysis and inducing apoptosis in both A549 and H1299 cell lines [152]. Similarly, Jin et al. suggest that p21 enhances glycolysis under hypoxic conditions via the transcriptional factor Hypoxia-Inducible Factor-1 alpha (HIF-1α), a factor known to regulate the transcriptional activation of a broad range of genes, facilitating the adaptation of tumor cells to hypoxic environments. Such genes include those that encode the enzymes GLUT1 and LDHA, responsible for enhancing glycolysis [153]. However, Chen et al. reported that Chromobox protein homolog 3 (CBX3) knockdown, mediated by the p53/p21 pathway, reduces the glycolysis in ovarian cancer cells [154]. While the evidence is compelling, it remains circumstantial, and more mechanistic studies are needed to elucidate the specific mechanisms by which p21 regulates glycolysis.

### 7.2. p21 and AMPK

Recent evidence indicates that p21’s involvement in metabolic stress may be related to the AMP-activated protein kinase (AMPK) pathway. AMPK serves as a highly sensitive sensor of cellular energy status, activated when there is a reduction in ATP production, often accompanied by an elevation in AMP and ADP levels [155]. Once activated, AMPK stimulates catabolic pathways to enhance ATP production while simultaneously suppressing anabolic ones [155]. Additionally, AMPK phosphorylates and activates p53 transcription, leading to the upregulation of p21 transcription, subsequently inducing cell cycle arrest from the G1 to the S phase [156]. Notably, a reduction in the ATP to AMP/ADP ratio has been observed during nutrient deprivation, particularly glucose deprivation, indicating that the glucose levels are involved in cell cycle progression through the AMPK-p21 pathway.

It is plausible that AMPK could modulate p21 by regulating ATP consumption in anabolic pathways or adjusting glycolytic flux patterns. However, it is unclear whether AMPK affects p21 directly by interacting with downstream pathways or indirectly through its connection to metabolic stress. Further mechanistic investigations would provide insight into the nature and directionality of this relationship.

### 7.3. p21 and RAS/RAF/MEK/ERK Pathway in Cancer Metabolism

The landscape of cancer research has been deeply influenced by the role of RAS mutations and the RAS pathway, which collectively exert a critical influence on cell cycle regulation and responses to growth signals [157,158,159]. Excessive RAS signaling triggers cell cycle arrest and senescence through the activation of RAF/ERK signaling. This phenomenon underscores the delicate equilibrium that exists between stimulating cell proliferation and inducing a state of growth arrest [160,161,162].

In specific types of cancer cells, the increase in p21 is linked to the actions of the enzyme I isoprenylcysteine carboxylmethyltransferase (ICMT) [163,164]. Evidence from mouse models of RAS tumorigenesis indicates that the loss of ICMT function causes the elevation of p21 levels, establishing a discernible link between the RAS pathway and p21 expression in orchestrating cellular processes [165,166,167,168].

**Figure 2:**
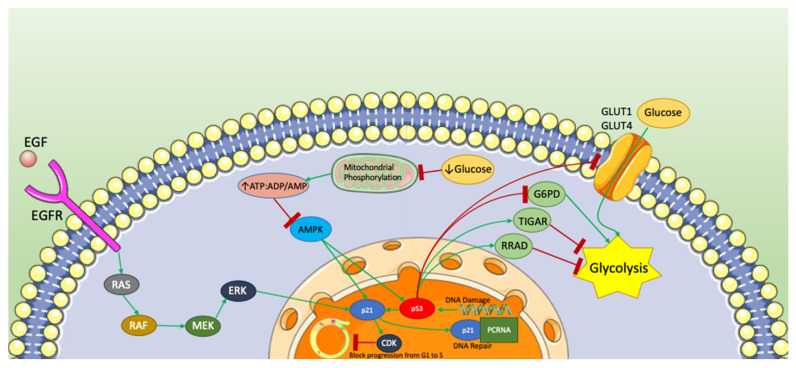
Summary of various metabolic pathways involving p21. A schematic showing p21’s central role in multiple biological pathways. In the RAS/RAF/MEK/ERK cascade, activation triggers p21 to bind with cyclin-dependent kinases (CDKs), exerting inhibitory control over the cell cycle. Metabolic stress, including low glucose levels, influences p21 modulation, ultimately leading to the upregulation of both p53 and p21, resulting in cell cycle arrest. p21 actively participates in anti-oncogenic pathways by binding with PCNA for DNA repair. The diagram illustrates p21 and p53’s interplay, with p53 sensing oncogenic processes and p21 acting as a cycle progression switch. The RAS/RAF/MEK/ERK pathway was observed in mice embryonic stem cells [165]. The AMPK pathway was observed in human embryonic kidney cells [156]. The p53/TIGAR pathway was observed in various human tumor cells including breast, lung, and bone tumor (osteosarcoma) cells [146]. The p53/RRAD pathway was observed in human lung cancer cells [147]. The p53/GLUT pathway was observed in human bone tumor (osteosarcoma) cells [150]. The p53/G6PD pathway was observed in human lung cancer cells [151]. EGF: EGF (Epidermal Growth Factor),]; EGFR: EGFR (Epidermal Growth Factor Receptor); *RAS: KRAS, HRAS, NRAS* (various RAS Proto-Oncogenes); *RAF*: *RAF1* (RAF1 Proto-Oncogene, Serine/Threonine Kinase); MEK: MAP2K1 (Mitogen-Activated Protein Kinase Kinase 1); ERK: MAPK1 (Mitogen-Activated Protein Kinase 1); ATP:ADP/AMP: ATP1A1; AMPK: PRKAA1, PRKAA2 (Protein Kinase AMP-Activated Catalytic Subunit Alpha 1 and 2); GLUT1: SLC2A1 (Solute Carrier Family 2 Member 1); GLUT4: SLC2A4 (Solute Carrier Family 2 Member 4); G6PD: G6PD (glucose-6-phosphate dehydrogenase); TIGAR: C12orf5 (TP53-Induced Glycolysis Regulatory Phosphatase); RRAD: RRAD (RAS-related glycolysis inhibitor and calcium channel regulator); PCRNA: PCNA (Proliferating Cell Nuclear Antigen).

Studying the effects of inhibiting ICMT, significant changes in cellular metabolism were observed. The cells sensitive to ICMT inhibition experienced a series of metabolic shifts, including reduced mitochondrial respiration, intensified autophagy, cell cycle arrest, and apoptosis [169,170,171]. Essentially, the RAS-associated pathways, p21, and the metabolism converged to propel the processes contributing to malignant transformation [166,167,168]. Figure 2 summarizes the metabolic pathways involving p21.

### 7.4. p21 and Autophagy

Cells are programmed to downregulate anabolic activity and upregulate autophagy to promote survival in response to metabolic stress [112]. This can be observed in diabetes, where the β cells in islets are exposed to very high levels of glucose. Yao et al. reported an increase in the autophagic activity in the β cells isolated from the subjects with T2DM. Furthermore, the viability of these β cells was significantly reduced after the knockdown of autophagy. These results suggest that autophagy plays a crucial role in protecting the cells from death and prolonging their insulin secretory function [172].

The direct involvement of p21 in the regulation of autophagy is yet to be thoroughly investigated. Nevertheless, numerous signaling molecules regulated by p21, such as Ask1, Gadd45, galectin-3, and prosaposin, have been implicated in the modulation of autophagy. Augmented autophagic activity was demonstrated in the human colorectal carcinoma cells (HCT1160) lacking p21 compared to the control group, confirming the inhibitory role of p21 in cellular autophagy. Also, the suppression of p21 is directly associated with elevated levels of Atg7, an essential enzyme involved in autophagy execution [173,174].

Recent evidence also indicates that Atg5, essential to the induction of autophagy, plays a regulatory role in H_2_O_2_-induced senescence by upregulating the expression of p21 [175]. This suggests that autophagy can activate p21, which in turn, promotes senescence.

## 8. p21 and the Efficacy of Diabetes Treatment

Diabetes is associated with a well-defined range of complications, such as nephropathy, retinopathy, peripheral neuropathy, cardiovascular diseases, delayed wound healing, and fertility issues [176]. Alongside its role in diabetes risk and severity, p21 is actively involved in these complications through the regulation of cell cycle arrest and senescence [177,178]. Targeting p21 as a potential treatment for diabetes offers the added advantage of addressing both diabetes and its related complications.

p21 is the most prominent effector molecule of the p53 target gene and has been shown to protect hypertrophied adipocytes from undergoing apoptosis in high fat diet-induced obese mice [179]. The upregulation of the p53/p21 axis in adipocytes by the retinoid X receptor (RXR) antagonist HX531 inhibits cellular hypertrophy and hyperplasia leading to cell cycle arrest in the G0/G1 stage. This leads to a reduction in fat pad mass, ameliorating the effects of obesity and diabetes [20]. RXR heterodimers and homodimers commonly target p21 [180], and treatment with HX531 reduces the binding of RXRα to RXRE’s upstream of the *CDKN1A* gene, also indicating that the up-regulation of this gene is not regulated by RXRs [181].

One study by Molnar et al. investigated the protective effects of metformin, a first line T2DM drug, and rapamycin, a macrolide, against diabetic nephropathy. The pathogenesis of diabetic nephropathy is attributed to cell proliferation and hypertrophy in the kidneys, primarily driven by cell cycle arrest and senescence [156]. The study showed that metformin protects against diabetic nephropathy by inhibiting p21, thereby preventing cell cycle arrest and offering renal protection. These effects were observed in human embryonic kidney cells. The study also revealed that increased glucose intake reverses the protective effects of metformin, inducing p21 expression and cell cycle arrest via the AMPK pathway [156].

Another treatment that exploits the role of p21 in inhibiting cellular hypertrophy and hyperplasia is ionizing radiation, which causes DNA damage and induces the p53 protein. p53 is phosphorylated after fibroblasts undergo apoptosis and senescence, thereby causing an increase in p21 expression [182]. However, cells that express mutant p53 are less sensitive to radiation and show prolonged induction of p21. p53-dependent cellular senescence was induced in human prostatic cells using ionizing radiation, revealing that the cells expressing mutant p53 had elevated p21 levels [183]. Furthermore, the accumulation of p21 depends on the p53/p21 axis, with mutant p53 acting as a substrate of DNA damage-induced protein kinases. The accumulation of mutant p53 due to Ser15 phosphorylation transactivates the p21 target gene [183]. Additionally, inducing the expression of p21 in an H1299 cell line also protects against the cytotoxic effects of ionizing radiation typically produced by double-strand breaks [184].

In a similar manner, genotoxic drugs lead to the activation of p53 in response to DNA damage. Several drugs fall under this category, and they exert their effect through the activation of p53. For example, doxorubicin is an anthracycline antibiotic derived from the *Streptomyces peucetius* bacterium [185]. Different concentrations of doxorubicin can produce different effects on DNA-damaged cells. Low concentrations of doxorubicin induce senescence by increasing the levels of p53, p21 and cyclin D1 [186]. The expression of p21 protects against the cytotoxic effects of doxorubicin, increasing senescence and decreasing apoptosis [185]. Conversely, apoptosis is characterized by low and prolonged p53 expression, the upregulation of E2F1, and the absence of p21 [187].

Another viable option for diabetes treatment is p53 gene therapy, where adenoviral vectors are used to transfer the wild-type version of the gene, allowing its direct expression. This type of gene therapy has previously been used for the treatment of lung cancer, preventing tumor formation [188]. p53 gene therapy induces autophagy and suppresses p21, which is associated with improvement in insulin resistance as reported in recent mice studies [124]. p21Cip1-highly expressing cells express SASP genes [189], which are closely correlated with insulin resistance [94]. Cell death is brought about by inducing ROS and depleting glutathione [190,191]. As such, the generation of ROS prevents senescence, which subsequently prevents insulin resistance [124]. In a related context, treatment with Gendicine has also been shown to induce the suppressive effects of p53 on glucose metabolism, as has been observed in cancer patients with insulin-dependent T2DM, with the effects persisting for over a year [192].

Given the strong relationship between oxidative activity and senescence downstream of *CDKN1A* [125], as first shown by Kaneto et al. [101], p21 gene therapy can be employed to induce ROS production in cancer cells by transfecting adenoviral vectors. Depending on the level of ROS induction, this can result in either apoptosis or senescence. By optimizing this relationship, it becomes possible to eliminate the senescent cells that contribute to the secretion of pro-inflammatory cytokines and chemokines, and which therefore provoke the deterioration of pancreatic β-cells. This approach aligns with recent strategies utilizing serotherapeutic principles in addressing T2DM [193].

A novel approach focuses upon the elimination of senescent cells, a process known as senotherapy, to treat metabolic disorders such as diabetes. The increase in fat mass seen due to an increase in the cell counts and the hypertrophy of the adipocytes [194], as well as a decline in adipogenesis, predisposes individuals over time to develop T2DM [195]. This is coupled with the senescence of white adipose tissue (WAT) in and around abdominal organs. Interestingly, the transplantation of WAT from the obese mice to the control mice induced insulin resistance, drawing a clear link between WAT senescence and diabetes [13].

Research involving mice has shown the involvement of both p16^Ink4^ and p21^Cip1^ in WAT senescence, specifically emphasizing the role of the p53/p21 axis in protecting against fat senescence [13,196,197]. Further research revealed that, while both the p16^Ink4^ and p21^Cip1^ levels increased in murine WAT, p21^Cip1^ elevation occurred earlier after the initiation of a high-fat diet, raising questions about its relative significance in senescence [94]. Moreover, targeting p21^high^ cells through senolysis suppresses the expression of SASP and genes, and improves insulin sensitivity [138].

As such, senolysis has been proposed as a candidate for the future management and potential treatment of diabetes. One of the first proposed drug combinations is dasatinib, a drug traditionally used to treat cancer [198], and quercetin, a natural flavanol [199], which successfully cleared senescent cells [138]. One study by Peng and colleagues found that dasatinib-induced senescence in ^KI^BRAF NSCLC cells was dependent on the accumulation of p21 [200]. Meanwhile, Ranelletti and colleagues previously demonstrated that quercetin inhibits p21-RAS expression in primary colorectal tumors and colon cancer cell lines [201]. The adverse effects associated with this combination are significant, however, and include fluid retention, hematologic dysfunction, skin rashes, and the prolongation of the QT interval [202].

A summary of the key studies regarding p21 in diabetes and related metabolic disorders is shown in Table 2.

## 9. Conclusions and Future Perspectives

Based on the evidence presented in this review, it is evident that p21 plays a central role in metabolic disorders and the severity of diabetic phenotypes. The involvement of p21 in cell cycle regulation and its participation in cellular senescence has been shown to exacerbate the development of metabolic disorders.

The regulation of p21 in various cell types in both animal and human subjects has emerged as a pivotal factor in the development of diabetic phenotypes. In many instances, lowering p21 levels through pharmacological means has resulted in improvements in diabetic symptoms [94].

However, it is important to recognize the intricate nature of p21’s role in diabetes. In certain contexts, evidence suggests that p21 acts as an inhibitor of ER stress-associated tissue damage. Boosting p21 activity may prove advantageous for managing diabetes and potentially other conditions characterized by undesirable ER stress-related cell death [105].

Therefore, while there is substantial evidence hinting at the potential benefits of inhibiting p21 for diabetes management, it is too early to definitively recommend this approach. Instead, it might be more prudent to focus on identifying the key pathways and genes that either regulate p21 or are regulated by it. In this regard, in a diet-induced obesity mouse model, it was observed that the Thr55 phosphorylation of p21 by MPK38 promotes its nuclear translocation and inhibits the PPARγ transactivation necessary for adipogenesis, ultimately leading to the amelioration of metabolic disorder traits [203].

As the number of individuals with diabetes continues to rise globally, it is imperative to develop novel preventative measures and treatments for diabetes. T2DM, which occurs mostly due to modifiable risk factors, accounts for 90% of the cases. Accumulating evidence suggests that further investigating the role of p21 in the development of diabetes may introduce new avenues of treatment and management.

## Figures and Tables

**Table 1:** Protein interactions of p21 and their binding regions. Overview of protein–protein interaction networks of p21, showing the proteins that interact with p21 and the regions of p21 responsible for binding. p21 binding regions refer to amino acid positions within the protein sequence. Cyclins: cyclins (Regulatory Proteins for Cell Cycle), CDKs: CDKs (cyclin-dependent kinases), PCNA: PCNA (Proliferating Cell Nuclear Antigen), ASK1: ASK1 (Apoptosis Signal-Regulating Kinase 1), C8 α-subunit of 20S proteasome: C8 α-subunit of 20S proteasome (Proteasome Complex Component), Calmodulin: Calmodulin (Calcium-Binding Messenger Protein), CARB: CARB (Cellular Apoptosis Susceptibility Protein), CK2: CK2 (Casein Kinase 2), c-Myc: c-Myc (Myc Proto-Oncogene Protein), E2F-1: E2F-1 (E2F Transcription Factor 1), GADD45: GADD45 (Growth Arrest and DNA Damage-Inducible Protein), MDM2: MDM2 (Mouse Double Minute 2), Procaspase 3: Procaspase 3 (Inactive Caspase 3 Precursor), SAPK: SAPK (Stress-Activated Protein Kinase), SET: SET (Multifunctional Protein in Chromatin Structure and Apoptosis), STAT3: STAT3 (Signal Transducer and Activator of Transcription 3), TOK1: TOK1 (Potassium Channel Protein), TSG101: TSG101 (Tumor Susceptibility Gene 101), WISp39: WISp39 (Heat Shock Protein Co-Chaperone), Cables1: Cables1 (CDK5 and Abl Enzyme Substrate 1).

Proteins	p21 Binding Regions	References
Cyclins	17–24 and 155–7	[38,39,40]
CDKs	53–8 and 74–9	[38,39,40]
PCNA	143–60	[38,39,40]
ASK1	1–140	[41]
C8 α-subunit of 20S proteasome	140–64	[42]
Calmodulin	145–64	[43,44]
CARB	Not determined	[45]
CK2	46–65	[46,47,48]
c-Myc	139–64	[49]
E2F-1	1–90	[50]
GADD45	139–64	[51,52]
MDM2	87–164	[53]
Procaspase 3	1–33	[54,55]
SAPK	1–84	[56]
SET	140–4 and 156–64	[57]
STAT3	Not determined	[58]
TOK1	149–64	[59]
TSG101	1–86	[60]
WISp39	28–56	[61]
Cables1	46–89	[62]

**Table 2:** A summary of the key studies regarding p21 in diabetes and related metabolic disorders.

Author/Year	Title	Method of Study	Cell Type	Main Relevant Findings
Kaneto. et al. [101]	Oxidative stress induces p21 expression in pancreatic islet cells: possible implication in beta-cell dysfunction	Oxidative stress was induced in isolated rat pancreatic islet cells by treatment with hydrogen peroxide	Rat pancreatic islet cells	Oxidative stress triggers the upregulation of cyclin-dependent kinase inhibitor p21 in pancreatic islet cells, a response that becomes more pronounced with diabetes progression. This p21 activation likely plays a role in beta-cell glucose toxicity by limiting cell proliferation and impairing insulin synthesis. This was noted when the overexpression of p21 was accompanied by the suppression of insulin mRNA in the isolated islets cells.
Mihailidou et al. [102]	The regulation of P21 during diabetes-associated stress of the endoplasmic reticulum	Examined p21 activity during ER stress and how is it regulated in the context of diabetes	Hamster pancreatic islet β-cell line	p21 can be utilized to adjust cell sensitivity to ER stress, which can reduce apoptosis and improve outcomes in diabetes.
Zhang et al. [105]	The cytotoxic role of intermittent high glucose on apoptosis and cell viability in pancreatic beta cells	Cells were treated with normal glucose (5.5 mmol/L), constant high glucose (CHG) (25 mmol/L), and IHG (rotation per 24 h in 11.1 or 25 mmol/L) for 7 days	INS-1 cells	Chronic exposure to intermittent high glucose will lead to effective induction of apoptosis by increasing the intracellular oxidative stress produced by hyperactivated xanthine oxidase activity.
Inoue et al. [131]	Cyclin-dependent kinase inhibitor, p21WAF1/CIP1, is involved in adipocyte differentiation and hypertrophy, linking to obesity, and insulin resistance	3T3-L1 fibroblasts were differentiated into adipocytes, and p21 expression was assessed. p21waf1/Cip1 knock-out mice were either maintained on a normal chow diet or an obesity-induced diet with a high fat high sucrose (HFHS) diet with weekly collections of their blood for metabolic analysis (measurement of blood glucose, insulin, triglyceride, total cholesterol, and free fatty acid) and measured body weight.	3T3-L1 cells and p21waf1/Cip1 knock-out mice	p21 prevents the apoptosis of hypertrophic adipocytes and increasing obesity is linked to insulin resistance. When p21is absent, adipose tissue expansion is suppressed, and obesity is ameliorated.
Wang et al. [94]	Targeting p21(Cip1) highly expressing cells in adipose tissue alleviates insulin resistance in obesity	2-month-old male C57BL/6 mice were given either regular chow diet or high-fat diet for 2 months, and single-cell transcriptomic (SCT) analysis was performed. SCT information from 11,401 and 7283 cells from lean and obese mice, respectively, was collected.	p21high cells of mice adipose tissue	Intermittent clearance of p21-high cells can prevent and alleviate insulin resistance in obese mice. Inactivation of NF-κB pathway within p21high cells helps reduce insulin resistance. Also, p21high cells within fat are sufficient to cause insulin resistance in vivo.
Hernandez et al. [86]	Upregulation of p21 activates the intrinsic apoptotic pathway in beta-cells	INS-1-derived 832/13 cells were applied pharmacological stress by dexamethasone and thapsigargin. P21 overexpression was assessed by [3H]methyl-thymidine incorporation. Cell cycle analysis and apoptosis analysis was performed by flow cytometry.	INS-1-derived 832/13 and 828/33 rat insulinoma cells	Cellular stress impairs β-cell proliferation and induces apoptosis which leads to insulin secretion reduction and can make it harder to control glucose homeostasis in insulin resistance or type 2 diabetes
Yang et al. [134]	P21cip overexpression in the mouse β Cells leads to improved recovery from streptozotocin-induced diabetes	A novel transgenic mouse model was developed to study the pancreatic β cell regeneration, which could specifically inhibit β cell proliferation by overexpressing p21 cip in β cells via regulation of the Tet-on system.	Pancreatic mouse β cells	p21’s overexpression can have both detrimental and beneficial effects on β-cells, as it exacerbates glucotoxicity-induced apoptosis while also promoting β-cell recovery.
Tinkum et al. [141]	Forkhead box O1 (FOXO1) protein, but not p53, contributes to the robust induction of p21 expression in fasted mice	Low-light bioluminescent imaging was employed to localize p21 expression to specific regions of the brain, which enabled the examination of p21 expression under short-term starvation (fasting)	In vivo reporter mice	Short-term fasting has been shown to increase p21 expression in mouse organs including the brain.
Lopez-Guadamillas et al. [142]	p21Cip1 plays a critical role in the physiological adaptation to fasting through activation of PPARα	Mice were fed with standard chow diet or fasted for 48 or 24 h. Primary hepatocytes were extracted and an RNA analysis was performed. Serum analysis was also performed.	Primary hepatocytes from mice	Only p21 mRNA is upregulated in fasting, being more prominent in the liver and muscle, while p16Ink4a, p19Arf, p27Kip1, and p53 mRNAs are unaffected.
Chu et al. [152]	miR-512-5p induces apoptosis and inhibits glycolysis by targeting p21 in non-small cell lung cancer cells	miR-512-5p was induced and inhibited in the cells, and the subsequent cell proliferation, apoptosis, glucose consumption, and lactate production were measured.	Non-small cell lung carcinoma cells	The overexpression of miR-512-5p induced apoptosis in non-small cell lung cancer (NSCLC) cells, while inhibiting glycolysis and migration. *CDKN1A* was identified as a target gene of miR-512-5p, with its overexpression leading to a decrease in both the p21 protein and mRNA levels. The knockdown of p21 mimicked the effects of miR-512-5p overexpression, including enhanced apoptosis and reduced glycolysis, and also counteracted the inhibitory effect of miR-512-5p on cell apoptosis.
Jin et al. [153]	A positive feedback circuit comprising p21 and HIF-1α aggravates the hhypox-ia-induced radioresistance of glioblastoma by promoting Glut1/LDHA-mediated glycolysis	The cells were examined under hypoxic conditions and p21/HIF-1α and related genes were measured. The cells were also exposed to radiation and the same parameters were measured.	Human glioblastoma and human glioma cells	p21 is directly activated at the transcriptional level by HIF-1α, which subsequently enhances the transcription of HIF-1α itself. This results in the upregulation of HIF-1α-responsive genes, including glycolysis-related enzymes like Glut1 and LDHA, thereby promoting glycolysis. Increased glycolysis, in turn, contributes to the radioresistance of glioblastoma multiforme (GBM) through various molecular mechanisms.
Chen et al. [154]	CBX3 promotes ovarian cancer progression by regulating p53/p21-mediated glucose metabolism via inhibiting NCOR2	The expression of Chromobox protein homolog 3 (CBX3) was analyzed in ovarian cancer cells, along with its effects on cell proliferation, cell cycle regulation, and apoptosis. Additionally, the modulatory influence of CBX3 on NCOR2 expression and p53/p21-mediated glycolysis was evaluated.	Epithelial ovarian cancer tumor cells and corresponding adjacent non-tumor cells	CBX3 was significantly overexpressed in the ovarian cancer (OC) tissues and cell lines, showing a negative correlation with NCOR2. It enhanced the viability, migration, and invasion of the OC cells by activating p53/p21-mediated glycolysis through the inhibition of NCOR2.
Molnar et al. [156]	p21WAF1/CIP1 expression is differentially regulated by metformin and rapamycin	The cells were treated with varying doses of metformin and rapamycin, and the effects on p21 expression, AMPK activity, and cell cycle senescence were assessed. These results were compared to untreated control cells. Additionally, the drug-treated cells were exposed to a high-glucose environment to evaluate its impact on these parameters	Human embryonic kidney (HEK293) cell	This study demonstrates that metformin suppresses high glucose-induced p21 expression. High glucose promotes cell cycle senescence through p21, a recognized mechanism in the pathophysiology of diabetic nephropathy. Metformin was found to counteract this effect, mediated by specific AMPK isoforms.
Maheshwari et al. [174]	Inhibition of p21 activates Akt kinase to trigger ROS-induced autophagy and impacts on tumor growth rate	The measurement of p21/akt and various genes were measured in human colorectal cells in comparison to the control cells	Human colorectal cancer cells	This study reveals that p21 plays a role in suppressing basal autophagy at physiological levels. When p21 is reduced, the activation of Akt appears to be the key mechanism driving the changes in autophagy regulation. Furthermore, p21 functions as a natural inhibitor of autophagy and exhibits oncogenic properties during the early phases of tumorigenesis.
Cmielová et al. [182]	DNA damage caused by ionizing radiation in embryonic diploid fibroblasts WI-38 induces both apoptosis and senescence	Ionizing radiation was introduced to the human embryonic lung diploid fibroblasts; the p53 and p21 activity were subsequently measured	WI-38 cells (human embryonic lung diploid fibroblasts)	Upon exposure to ionizing radiation, p53 becomes activated, which subsequently triggers the activation of its downstream target, p21. This leads to the activation of CDK inhibitors, inducing cell cycle arrest. This highlights p21’s critical role in mediating the effects of radiation exposure that kill cancer cells.

## Data Availability

Not applicable.

## Abbreviations

**Abbreviation**	**Definition**
AMP	Adenosine Monophosphate
AMPK	AMP-Activated Protein Kinase
AP2	Activator Protein 2
ASK1	Apoptosis Signal-Regulating Kinase 1
ATP	Adenosine Triphosphate
ATP1A1	ATPase Na+/K+ Transporting Subunit Alpha 1
BAX	BCL2-Associated X Protein
BAK	BCL2 Antagonist/Killer 1
BRCA1	Breast Cancer 1
BRCA2	Breast Cancer 2
CDK	Cyclin-Dependent Kinase
CDKN1A	Cyclin-Dependent Kinase Inhibitor 1A
CHOP	C/EBP Homologous Protein
CK2	Casein Kinase 2
CREBBP	CREB-Binding Protein
CRD1	C-Terminal Sumoylation Domain
DNA	Deoxyribonucleic Acid
DREAM	Dp, Retinoblastoma (Rb), E2F, and MuvB Complex
E2F	E2F Transcription Factor
ER	Endoplasmic Reticulum
ERK	Extracellular Signal-Regulated Kinase
FOXO1	Forkhead Box Protein O1
GADD45	Growth Arrest and DNA Damage-Inducible Protein
G6PD	Glucose-6-Phosphate Dehydrogenase
GLUT1	Glucose Transporter 1
GLUT4	Glucose Transporter 4
HCT116	Human Colorectal Carcinoma Cell Line
HIF-1α	Hypoxia-Inducible Factor 1-Alpha
IAPP	Islet Amyloid Polypeptide
ICMT	Isoprenylcysteine Carboxyl Methyltransferase
IDF	International Diabetes Federation
IGF-I	Insulin-Like Growth Factor I
IRS-1	Insulin Receptor Substrate 1
KLF10	Kruppel-Like Factor 10
KLF4	Kruppel-Like Factor 4
LDHA	Lactate Dehydrogenase A
MAPK	Mitogen-Activated Protein Kinase
MEK	Mitogen-Activated Protein Kinase Kinase
MDM2	Mouse Double Minute 2 Proto-Oncogene
NF-κB	Nuclear Factor Kappa-Light-Chain-Enhancer of Activated B Cells
NLS	Nuclear Localization Signal
PCNA	Proliferating Cell Nuclear Antigen
PKB	Protein Kinase B
PUMA	p53 Upregulated Modulator of Apoptosis
RAS	Rat Sarcoma Virus Proto-Oncogene
RAF	Rapidly Accelerated Fibrosarcoma Proto-Oncogene
RRAD	Ras-Related Glycolysis Inhibitor and Calcium Channel Regulator
SAPK	Stress-Activated Protein Kinase
SASP	Senescence-Associated Secretory Phenotype
STAT3	Signal Transducer and Activator of Transcription 3
T2DM	Type 2 Diabetes Mellitus
TGF-β	Transforming Growth Factor Beta
TIGAR	TP53-Induced Glycolysis and Apoptosis Regulator
TOK1	Potassium Transporter TOK1
UPR	Unfolded Protein Response
VAT	Visceral Adipose Tissue
VSMC	Vascular Smooth Muscle Cells
WISP3	WNT1-Inducible Signaling Pathway Protein 3

## References

[1] Sun H., Saeedi P., Karuranga S., Pinkepank M., Ogurtsova K., Duncan B.B., Stein C., Basit A., Chan J.C.N., Mbanya J.C. (2022). IDF Diabetes Atlas: Global, regional and country-level diabetes prevalence estimates for 2021 and projections for 2045. Diabetes Res. Clin. Pract..

[2] GBD 2021 Diabetes Collaborators (2023). Global, regional, and national burden of diabetes from 1990 to 2021, with projections of prevalence to 2050: A systematic analysis for the Global Burden of Disease Study 2021. Lancet.

[3] Hubbard S.R., Wei L., Ellis L., Hendrickson W.A. (1994). Crystal structure of the tyrosine kinase domain of the human insulin receptor. Nature.

[4] Ota T. (2014). Obesity-Induced Inflammation and Insulin Resistance. Front. Endocrinol..

[5] Yaribeygi H., Farrokhi F.R., Butler A.E., Sahebkar A. (2019). Insulin resistance: Review of the underlying molecular mechanisms. J. Cell. Physiol..

[6] Jones R.G., Plas D.R., Kubek S., Buzzai M., Mu J., Xu Y., Birnbaum M.J., Thompson C.B. (2005). AMP-activated protein kinase induces a p53-dependent metabolic checkpoint. Mol. Cell.

[7] Deng C., Zhang P., Harper J.W., Elledge S.J., Leder P. (1995). Mice lacking p21CIP1/WAF1 undergo normal development, but are defective in G1 checkpoint control. Cell.

[8] Cazzalini O., Perucca P., Savio M., Necchi D., Bianchi L., Stivala L.A., Ducommun B., Scovassi A.I., Prosperi E. (2008). Interaction of p21(CDKN1A) with PCNA regulates the histone acetyltransferase activity of p300 in nucleotide excision repair. Nucleic Acids Res..

[9] He L., Chen Y., Feng J., Sun W., Li S., Ou M., Tang L. (2017). Cellular senescence regulated by SWI/SNF complex subunits through p53/p21 and p16/pRB pathway. Int. J. Biochem. Cell Biol..

[10] Reinhardt H.C., Schumacher B. (2012). The p53 network: Cellular and systemic DNA damage responses in aging and cancer. Trends Genet..

[11] Gorospe M., Wang X., Holbrook N.J. (1999). Functional role of p21 during the cellular response to stress. Gene Expr..

[12] Gorospe M., Holbrook N.J. (1996). Role of p21 in prostaglandin A2-mediated cellular arrest and death. Cancer Res..

[13] Minamino T., Orimo M., Shimizu I., Kunieda T., Yokoyama M., Ito T., Nojima A., Nabetani A., Oike Y., Matsubara H. (2009). A crucial role for adipose tissue p53 in the regulation of insulin resistance. Nat. Med..

[14] Zhang L., Chen Z., Wang Y., Tweardy D.J., Mitch W.E. (2020). Stat3 activation induces insulin resistance via a muscle-specific E3 ubiquitin ligase Fbxo40. Am. J. Physiol. Endocrinol. Metab..

[15] Tze-chen H., Barbara B.D., Joseph M.W., Farid A.B. (2018). c-Myc Metabolic Addiction in Cancers Counteracted by Resveratrol and NQO2. Resveratrol.

[16] Denechaud P.D., Fajas L., Giralt A. (2017). E2F1, a Novel Regulator of Metabolism. Front. Endocrinol. (Lausanne).

[17] Laptenko O., Beckerman R., Freulich E., Prives C. (2011). p53 binding to nucleosomes within the p21 promoter in vivo leads to nucleosome loss and transcriptional activation. Proc. Natl. Acad. Sci. USA.

[18] Hardie D.G. (2011). AMP-activated protein kinase: An energy sensor that regulates all aspects of cell function. Genes Dev..

[19] McCubrey J.A., Steelman L.S., Chappell W.H., Abrams S.L., Wong E.W., Chang F., Lehmann B., Terrian D.M., Milella M., Tafuri A. (2007). Roles of the Raf/MEK/ERK pathway in cell growth, malignant transformation and drug resistance. Biochim. Biophys. Acta.

[20] Al Bitar S., Gali-Muhtasib H. (2019). The Role of the Cyclin Dependent Kinase Inhibitor p21^cip1/waf1^ in Targeting Cancer: Molecular Mechanisms and Novel Therapeutics. Cancers.

[21] Follis A.V., Galea C.A., Kriwacki R.W. (2012). Intrinsic protein flexibility in regulation of cell proliferation: Advantages for signaling and opportunities for novel therapeutics. Adv. Exp. Med. Biol..

[22] Chen I.T., Akamatsu M., Smith M.L., Lung F.D., Duba D., Roller P.P., Fornace A.J., O’Connor P.M. (1996). Characterization of p21Cip1/Waf1 peptide domains required for cyclin E/Cdk2 and PCNA interaction. Oncogene.

[23] Gartel A.L., Tyner A.L. (1999). Transcriptional regulation of the p21((WAF1/CIP1)) gene. Exp. Cell Res..

[24] Gartel A.L., Radhakrishnan S.K. (2005). Lost in transcription: p21 repression, mechanisms, and consequences. Cancer Res..

[25] Jung Y.S., Qian Y., Chen X. (2010). Examination of the expanding pathways for the regulation of p21 expression and activity. Cell Signal..

[26] Warfel N.A., El-Deiry W.S. (2013). p21WAF1 and tumourigenesis: 20 years after. Curr. Opin. Oncol..

[27] Shamloo B., Usluer S. (2019). p21 in Cancer Research. Cancers.

[28] Yu F., Megyesi J., Safirstein R.L., Price P.M. (2005). Identification of the functional domain of p21(WAF1/CIP1) that protects cells from cisplatin cytotoxicity. Am. J. Physiol. Renal Physiol..

[29] Fotedar R., Fitzgerald P., Rousselle T., Cannella D., Dorée M., Messier H., Fotedar A. (1996). p21 contains independent binding sites for cyclin and cdk2: Both sites are required to inhibit cdk2 kinase activity. Oncogene.

[30] Chen J., Saha P., Kornbluth S., Dynlacht B.D., Dutta A. (1996). Cyclin-binding motifs are essential for the function of p21CIP1. Mol. Cell. Biol..

[31] Kreis N.N., Louwen F., Yuan J. (2019). The Multifaceted p21 (Cip1/Waf1/*CDKN1A*) in Cell Differentiation, Migration and Cancer Therapy. Cancers.

[32] Lu J., Wu T., Zhang B., Liu S., Song W., Qiao J., Ruan H. (2021). Types of nuclear localization signals and mechanisms of protein import into the nucleus. Cell Commun. Signal..

[33] Hinshaw D.C., Shevde L.A. (2019). The Tumor Microenvironment Innately Modulates Cancer Progression. Cancer Res..

[34] Agell N., Jaumot M., Rodríguez-Vilarrupla A., Brun S., Abella N., Canela N., Estanyol J.M., Bachs O. (2006). The diverging roles of calmodulin and PKC in the regulation of p21 intracellular localization. Cell Cycle.

[35] Child E.S., Mann D.J. (2006). The intricacies of p21 phosphorylation: Protein/protein interactions, subcellular localization and stability. Cell Cycle.

[36] Garcia-Wilson E., Perkins N.D. (2005). p21WAF1/CIP1 Regulates the p300 Sumoylation Motif CRD1 through a C-Terminal Domain Independently of Cyclin/CDK Binding. Cell Cycle.

[37] Kroker A.J., Bruning J.B. (2015). p21 Exploits Residue Tyr151 as a Tether for High-Affinity PCNA Binding. Biochemistry.

[38] Lee M.H., Yang H.Y. (2001). Negative regulators of cyclin-dependent kinases and their roles in cancers. Cell. Mol. Life Sci..

[39] Schwartz G.K. (2002). CDK inhibitors: Cell cycle arrest versus apoptosis. Cell Cycle.

[40] Coqueret O. (2003). New roles for p21 and p27 cell-cycle inhibitors: A function for each cell compartment?. Trends Cell Biol..

[41] Asada M., Yamada T., Ichijo H., Delia D., Miyazono K., Fukumuro K., Mizutani S. (1999). Apoptosis inhibitory activity of cytoplasmic p21(Cip1/WAF1) in monocytic differentiation. EMBO J..

[42] Touitou R., Richardson J., Bose S., Nakanishi M., Rivett J., Allday M.J. (2001). A degradation signal located in the C-terminus of p21WAF1/CIP1 is a binding site for the C8 alpha-subunit of the 20S proteasome. EMBO J..

[43] Rodríguez-Vilarrupla A., Jaumot M., Abella N., Canela N., Brun S., Díaz C., Estanyol J.M., Bachs O., Agell N. (2005). Binding of calmodulin to the carboxy-terminal region of p21 induces nuclear accumulation via inhibition of protein kinase C-mediated phosphorylation of Ser153. Mol. Cell. Biol..

[44] Taulés M., Rodríguez-Vilarrupla A., Rius E., Estanyol J.M., Casanovas O., Sacks D.B., Pérez-Payá E., Bachs O., Agell N. (1999). Calmodulin Binds to p21Cip1 and Is Involved in the Regulation of Its Nuclear Localization. J. Biol. Chem..

[45] McShea A., Samuel T., Eppel J.T., Galloway D.A., Funk J.O. (2000). Identification of CIP-1-associated regulator of cyclin B (CARB), a novel p21-binding protein acting in the G2 phase of the cell cycle. J. Biol. Chem..

[46] Götz C., Wagner P., Issinger O.G., Montenarh M. (1996). p21WAF1/CIP1 interacts with protein kinase CK2. Oncogene.

[47] Bertrand L., Sayed M.F., Pei X.Y., Parisini E., Dhanaraj V., Bolanos-Garcia V.M., Allende J.E., Blundell T.L. (2004). Structure of the regulatory subunit of CK2 in the presence of a p21WAF1 peptide demonstrates flexibility of the acidic loop. Acta Crystallogr. D Biol. Crystallogr..

[48] Götz C., Kartarius S., Scholtes P., Montenarh M. (2000). Binding domain for p21(WAF1) on the polypeptide chain of the protein kinase CK2 beta-subunit. Biochem. Biophys. Res. Commun..

[49] Krengel U., Schlichting I., Scherer A., Schumann R., Frech M., John J., Kabsch W., Pai E.F., Wittinghofer A. (1990). Three-dimensional structures of H-ras p21 mutants: Molecular basis for their inability to function as signal switch molecules. Cell.

[50] Delavaine L., La Thangue N.B. (1999). Control of E2F activity by p21Waf1/Cip1. Oncogene.

[51] Kearsey J.M., Coates P.J., Prescott A.R., Warbrick E., Hall P.A. (1995). Gadd45 is a nuclear cell cycle regulated protein which interacts with p21Cip1. Oncogene.

[52] Vairapandi M., Balliet A.G., Fornace A.J., Jr Hoffman B., Liebermann D.A. (1996). The differentiation primary response gene MyD118, related to GADD45, encodes for a nuclear protein which interacts with PCNA and p21WAF1/CIP1. Oncogene.

[53] Jin Y., Lee H., Zeng S.X., Dai M.S., Lu H. (2003). MDM2 promotes p21waf1/cip1 proteasomal turnover independently of ubiquitylation. EMBO J..

[54] Suzuki A., Tsutomi Y., Akahane K., Araki T., Miura M. (1998). Resistance to Fas-mediated apoptosis: Activation of caspase 3 is regulated by cell cycle regulator p21WAF1 and IAP gene family ILP. Oncogene.

[55] Suzuki A., Tsutomi Y., Miura M., Akahane K. (1999). Caspase 3 inactivation to suppress Fas-mediated apoptosis: Identification of binding domain with p21 and ILP and inactivation machinery by p21. Oncogene.

[56] Shim J., Lee H., Park J., Kim H., Choi E.J. (1996). A non-enzymatic p21 protein inhibitor of stress-activated protein kinases. Nature.

[57] Canela N., Rodriguez-Vilarrupla A., Estanyol J.M., Diaz C., Pujol M.J., Agell N., Bachs O. (2003). The SET protein regulates G2/M transition by modulating cyclin B-cyclin-dependent kinase 1 activity. J. Biol. Chem..

[58] Coqueret O., Gascan H. (2000). Functional interaction of STAT3 transcription factor with the cell cycle inhibitor p21WAF1/CIP1/SDI1. J. Biol. Chem..

[59] Ono T., Kitaura H., Ugai H., Murata T., Yokoyama K.K., Iguchi-Ariga S.M., Ariga H. (2000). TOK-1, a novel p21Cip1-binding protein that cooperatively enhances p21-dependent inhibitory activity toward CDK2 kinase. J. Biol. Chem..

[60] Oh H., Mammucari C., Nenci A., Cabodi S., Cohen S.N., Dotto G.P. (2002). Negative regulation of cell growth and differentiation by TSG101 through association with p21(Cip1/WAF1). Proc. Natl. Acad. Sci. USA.

[61] Jascur T., Brickner H., Salles-Passador I., Barbier V., El Khissiin A., Smith B., Fotedar R., Fotedar A. (2005). Regulation of p21(WAF1/CIP1) stability by WISp39, a Hsp90 binding TPR protein. Mol. Cell.

[62] Shi Z., Li Z., Li Z.J., Cheng K., Du Y., Fu H., Khuri F.R. (2015). Cables1 controls p21/Cip1 protein stability by antagonizing proteasome subunit alpha type 3. Oncogene.

[63] Koutsodontis G., Tentes I., Papakosta P., Moustakas A., Kardassis D. (2001). Sp1 plays a critical role in the transcriptional activation of the human cyclin-dependent kinase inhibitor p21(WAF1/Cip1) gene by the p53 tumor suppressor protein. J. Biol. Chem..

[64] Decesse J.T., Medjkane S., Datto M.B., Crémisi C.E. (2001). RB regulates transcription of the p21/WAF1/CIP1 gene. Oncogene.

[65] Abbas T., Dutta A. (2009). p21 in cancer: Intricate networks and multiple activities. Nat. Rev. Cancer.

[66] Xu H., Wang Z., Jin S., Hao H., Zheng L., Zhou B., Zhang W., Lv H., Yuan Y. (2014). Dux4 induces cell cycle arrest at G1 phase through upregulation of p21 expression. Biochem. Biophys. Res. Commun..

[67] Bai Y.Q., Miyake S., Iwai T., Yuasa Y. (2003). CDX2, a homeobox transcription factor, upregulates transcription of the p21/WAF1/CIP1 gene. Oncogene.

[68] Fang Z., Fu Y., Liang Y., Li Z., Zhang W., Jin J., Yang Y., Zha X. (2007). Increased expression of integrin beta1 subunit enhances p21WAF1/Cip1 transcription through the Sp1 sites and p300-mediated histone acetylation in human hepatocellular carcinoma cells. J. Cell. Biochem..

[69] Li D., Yea S., Dolios G., Martignetti J.A., Narla G., Wang R., Walsh M.J., Friedman S.L. (2005). Regulation of Kruppel-like factor 6 tumor suppressor activity by acetylation. Cancer Res..

[70] Elston R., Inman G.J. (2012). Crosstalk between p53 and TGF-β Signalling. J. Signal Transduct..

[71] Parveen A., Akash M.S., Rehman K., Kyunn W.W. (2016). Dual Role of p21 in the Progression of Cancer and Its Treatment. Crit. Rev. Eukaryot. Gene Expr..

[72] Vigneron A., Cherier J., Barré B., Gamelin E., Coqueret O. (2006). The cell cycle inhibitor p21waf1 binds to the myc and cdc25A promoters upon DNA damage and induces transcriptional repression. J. Biol. Chem..

[73] Chang B.D., Watanabe K., Broude E.V., Fang J., Poole J.C., Kalinichenko T.V., Roninson I.B. (2000). Effects of p21Waf1/Cip1/Sdi1 on cellular gene expression: Implications for carcinogenesis, senescence, and age-related diseases. Proc. Natl. Acad. Sci. USA.

[74] Engeland K. (2018). Cell cycle arrest through indirect transcriptional repression by p53: I have a DREAM. Cell Death Differ..

[75] Xiong Y., Hannon G.J., Zhang H., Casso D., Kobayashi R., Beach D. (1993). p21 is a universal inhibitor of cyclin kinases. Nature.

[76] Sapra A., Bhandari P. (2023). Diabetes. StatPearls.

[77] Butler A.E., Misselbrook D. (2020). Distinguishing between type 1 and type 2 diabetes. BMJ.

[78] Pirjani R., Shirzad N., Qorbani M., Phelpheli M., Nasli-Esfahani E., Bandarian F., Hemmatabadi M. (2017). Gestational diabetes mellitus its association with obesity: A prospective cohort study. Eat. Weight Disord..

[79] Hayfron-Benjamin C., van den Born B.J., Maitland-van der Zee A.H., Amoah A.G.B., Meeks K.A.C., Klipstein-Grobusch K., Bahendeka S., Spranger J., Danquah I., Mockenhaupt F. (2019). Microvascular and macrovascular complications in type 2 diabetes Ghanaian residents in Ghana and Europe: The RODAM study. J. Diabetes Complicat..

[80] Chen C., Cohrs C.M., Stertmann J., Bozsak R., Speier S. (2017). Human beta cell mass and function in diabetes: Recent advances in knowledge and technologies to understand disease pathogenesis. Mol. Metab..

[81] Cerf M.E. (2013). Beta cell dysfunction and insulin resistance. Front. Endocrinol. (Lausanne).

[82] Butler A.E., Janson J., Bonner-Weir S., Ritzel R., Rizza R.A., Butler P.C. (2003). Beta-cell deficit and increased beta-cell apoptosis in humans with type 2 diabetes. Diabetes.

[83] Saisho Y. (2015). β-cell dysfunction: Its critical role in prevention and management of type 2 diabetes. World J. Diabetes.

[84] Tomita T. (2016). Apoptosis in pancreatic β-islet cells in Type 2 diabetes. Bosn. J. Basic Med. Sci..

[85] Pucci B., Kasten M., Giordano A. (2000). Cell cycle and apoptosis. Neoplasia.

[86] Hernandez A.M., Colvin E.S., Chen Y.C., Geiss S.L., Eller L.E., Fueger P.T. (2013). Upregulation of p21 activates the intrinsic apoptotic pathway in beta-cells. Am. J. Physiol. Endocrinol. Metab..

[87] Fulda S., Gorman A.M., Hori O., Samali A. (2010). Cellular stress responses: Cell survival and cell death. Int. J. Cell Biol..

[88] Lee J.H., Lee J. (2022). Endoplasmic Reticulum (ER) Stress and Its Role in Pancreatic β-Cell Dysfunction and Senescence in Type 2 Diabetes. Int. J. Mol. Sci..

[89] Robertson R., Zhou H., Zhang T., Harmon J.S. (2007). Chronic oxidative stress as a mechanism for glucose toxicity of the beta cell in type 2 diabetes. Cell Biochem. Biophys..

[90] Vilas-Boas E.A., Almeida D.C., Roma L.P., Ortis F., Carpinelli A.R. (2021). Lipotoxicity and β-Cell Failure in Type 2 Diabetes: Oxidative Stress Linked to NADPH Oxidase and ER Stress. Cells.

[91] Quan W., Jo E.K., Lee M.S. (2013). Role of pancreatic β-cell death and inflammation in diabetes. Diabetes Obes. Metab..

[92] Butler A.E., Robertson R.P., Hernandez R., Matveyenko A.V., Gurlo T., Butler P.C. (2012). Beta cell nuclear musculoaponeurotic fibrosarcoma oncogene family A (MafA) is deficient in type 2 diabetes. Diabetologia.

[93] Moin A.S.M., Butler A.E. (2019). Alterations in Beta Cell Identity in Type 1 and Type 2 Diabetes. Curr. Diabetes Rep..

[94] Wang L., Wang B., Gasek N.S., Zhou Y., Cohn R.L., Martin D.E., Zuo W., Flynn W.F., Guo C., Jellison E.R. (2022). Targeting p21(Cip1) highly expressing cells in adipose tissue alleviates insulin resistance in obesity. Cell Metab..

[95] Ding L., Cao J., Lin W., Chen H., Xiong X., Ao H., Yu M., Lin J., Cui Q. (2020). The Roles of Cyclin-Dependent Kinases in Cell-Cycle Progression and Therapeutic Strategies in Human Breast Cancer. Int. J. Mol. Sci..

[96] Carnero A., Hannon G.J. (1998). The INK4 family of CDK inhibitors. Curr. Top. Microbiol. Immunol..

[97] Dutto I., Tillhon M., Cazzalini O., Stivala L.A., Prosperi E. (2015). Biology of the cell cycle inhibitor p21(CDKN1A): Molecular mechanisms and relevance in chemical toxicology. Arch. Toxicol..

[98] Paunesku T., Mittal S., Protić M., Oryhon J., Korolev S.V., Joachimiak A., Woloschak G.E. (2001). Proliferating cell nuclear antigen (PCNA): Ringmaster of the genome. Int. J. Radiat. Biol..

[99] Brugarolas J., Chandrasekaran C., Gordon J.I., Beach D., Jacks T., Hannon G.J. (1995). Radiation-induced cell cycle arrest compromised by p21 deficiency. Nature.

[100] Nakayama Y., Yamaguchi N. (2013). Role of cyclin B1 levels in DNA damage and DNA damage-induced senescence. Int. Rev. Cell Mol. Biol..

[101] Kaneto H., Kajimoto Y., Fujitani Y., Matsuoka T., Sakamoto K., Matsuhisa M., Yamasaki Y., Hori M. (1999). Oxidative stress induces p21 expression in pancreatic islet cells: Possible implication in beta-cell dysfunction. Diabetologia.

[102] Mihailidou C., Papazian I., Papavassiliou A.G., Kiaris H. (2010). CHOP-dependent regulation of p21/waf1 during ER stress. Cell Physiol. Biochem..

[103] Back S.H., Kaufman R.J. (2012). Endoplasmic reticulum stress and type 2 diabetes. Annu. Rev. Biochem..

[104] Mihailidou C., Chatzistamou I., Papavassiliou A.G., Kiaris H. (2015). Regulation of P21 during diabetes-associated stress of the endoplasmic reticulum. Endocr. Relat. Cancer.

[105] Zhang Z., Li J., Yang L., Chen R., Yang R., Zhang H., Cai D., Chen H. (2014). The cytotoxic role of intermittent high glucose on apoptosis and cell viability in pancreatic beta cells. J. Diabetes Res..

[106] Zhang S., Liu J., Saafi E.L., Cooper G.J. (1999). Induction of apoptosis by human amylin in RINm5F islet beta-cells is associated with enhanced expression of p53 and p21WAF1/CIP1. FEBS Lett..

[107] Hernandez-Carretero A. (2014). Novel Roles of p21 in Apoptosis During Beta-Cell Stress in Diabetes. Ph.D. Thesis.

[108] Karimian A., Ahmadi Y., Yousefi B. (2016). Multiple functions of p21 in cell cycle, apoptosis and transcriptional regulation after DNA damage. DNA Repair.

[109] Soria G., Speroni J., Podhajcer O.L., Prives C., Gottifredi V. (2008). p21 differentially regulates DNA replication and DNA-repair-associated processes after UV irradiation. J. Cell Sci..

[110] Kohli L., Roth K.A. (2010). Autophagy: Cerebral home cooking. Am. J. Pathol..

[111] Das G., Shravage B.V., Baehrecke E.H. (2012). Regulation and function of autophagy during cell survival and cell death. Cold Spring Harb. Perspect. Biol..

[112] Manu K.A., Cao P.H.A., Chai T.F., Casey P.J., Wang M. (2019). p21cip1/waf1 Coordinate Autophagy, Proliferation and Apoptosis in Response to Metabolic Stress. Cancers.

[113] Huang S., Xu M., Liu L., Yang J., Wang H., Wan C., Deng W., Tang Q. (2020). Autophagy is involved in the protective effect of p21 on LPS-induced cardiac dysfunction. Cell Death Dis..

[114] Vitiello P.F., Staversky R.J., Keng P.C., O’Reilly M.A. (2008). PUMA inactivation protects against oxidative stress through p21/Bcl-XL inhibition of bax death. Free Radic Biol. Med..

[115] Palmer A.K., Tchkonia T., LeBrasseur N.K., Chini E.N., Xu M., Kirkland J.L. (2015). Cellular Senescence in Type 2 Diabetes: A Therapeutic Opportunity. Diabetes.

[116] Narasimhan A., Flores R.R., Robbins P.D., Niedernhofer L.J. (2021). Role of Cellular Senescence in Type II Diabetes. Endocrinology.

[117] Sano R., Reed J.C. (2013). ER stress-induced cell death mechanisms. Biochim. Biophys. Acta.

[118] Corazzari M., Gagliardi M., Fimia G.M., Piacentini M. (2017). Endoplasmic Reticulum Stress, Unfolded Protein Response, and Cancer Cell Fate. Front. Oncol..

[119] Walter P., Ron D. (2011). The unfolded protein response: From stress pathway to homeostatic regulation. Science.

[120] Tay V.S.Y., Devaraj S., Koh T., Ke G., Crasta K.C., Ali Y. (2019). Increased double strand breaks in diabetic β-cells with a p21 response that limits apoptosis. Sci. Rep..

[121] Gurlo T., Rivera J.F., Butler A.E., Cory M., Hoang J., Costes S., Bulter P.C. (2016). CHOP Contributes to, But Is Not the Only Mediator of, IAPP Induced β-Cell Apoptosis. Mol. Endocrinol..

[122] Roninson I.B. (2002). Oncogenic functions of tumour suppressor p21(Waf1/Cip1/Sdi1): Association with cell senescence and tumour-promoting activities of stromal fibroblasts. Cancer Lett..

[123] Kumari R., Jat P. (2021). Mechanisms of Cellular Senescence: Cell Cycle Arrest and Senescence Associated Secretory Phenotype. Front. Cell Dev. Biol..

[124] Murakami T., Inagaki N., Kondoh H. (2022). Cellular Senescence in Diabetes Mellitus: Distinct Senotherapeutic Strategies for Adipose Tissue and Pancreatic beta Cells. Front. Endocrinol. (Lausanne).

[125] Storer M., Mas A., Robert-Moreno A., Pecoraro M., Ortells M.C., Di Giacomo V., Yosef R., Pilpel N., Krizhanovsky V., Sharpe J. (2013). Senescence is a developmental mechanism that contributes to embryonic growth and patterning. Cell.

[126] Aliu E., Kanungo S., Arnold G.L. (2018). Amino acid disorders. Ann. Transl. Med..

[127] Menge B.A., Schrader H., Ritter P.R., Ellrichmann M., Uhl W., Schmidt W.E., Meier J.J. (2010). Selective amino acid deficiency in patients with impaired glucose tolerance and type 2 diabetes. Regul. Pept..

[128] Leung-Pineda V., Pan Y., Chen H., Kilberg M.S. (2004). Induction of p21 and p27 expression by amino acid deprivation of HepG2 human hepatoma cells involves mRNA stabilization. Biochem. J..

[129] Rong Y., Darnell A.M., Sapp K.M., Vander Heiden M.G., Spencer S.L. (2023). Cells use multiple mechanisms for cell-cycle arrest upon withdrawal of individual amino acids. Cell Rep..

[130] Piché M.E., Tchernof A., Després J.P. (2020). Obesity Phenotypes, Diabetes, and Cardiovascular Diseases. Circ. Res..

[131] Inoue N., Yahagi N., Yamamoto T., Ishikawa M., Watanabe K., Matsuzaka T., Nakagawa Y., Takeuchi Y., Kobayashi K., Takahashi A. (2008). Cyclin-dependent kinase inhibitor, p21WAF1/CIP1, is involved in adipocyte differentiation and hypertrophy, linking to obesity, and insulin resistance. J. Biol. Chem..

[132] Wu M.J., Wu W.C., Chang H.W., Lai Y.T., Lin C.H., Yu W.C., Chang V.H. (2015). KLF10 affects pancreatic function via the SEI-1/p21Cip1 pathway. Int. J. Biochem. Cell Biol..

[133] Tornovsky-Babeay S., Dadon D., Ziv O., Tzipilevich E., Kadosh T., Schyr-Ben Haroush R., Hija A., Stolovich-Rain M., Furth-Lavi J., Granot Z. (2014). Type 2 diabetes and congenital hyperinsulinism cause DNA double-strand breaks and p53 activity in β cells. Cell Metab..

[134] Yang J., Zhang W., Jiang W., Sun X., Han Y., Ding M., Shi Y., Deng H. (2009). P21cip-Overexpression in the Mouse β Cells Leads to the Improved Recovery from Streptozotocin-Induced Diabetes. PLoS ONE.

[135] Ahn M., Yoder S.M., Wang Z., Oh E., Ramalingam L., Tunduguru R., Thurmond D.C. (2016). The p21-activated kinase (PAK1) is involved in diet-induced beta cell mass expansion and survival in mice and human islets. Diabetologia.

[136] Karamitopoulou E., Zlobec I., Tornillo L., Carafa V., Schaffner T., Brunner T., Borner M., Diamantis I., Zimmermann A., Terracciano L. (2010). Differential cell cycle and proliferation marker expression in ductal pancreatic adenocarcinoma and pancreatic intraepithelial neoplasia (PanIN). Pathology.

[137] Kim J.K., Kim Y.J., Fillmore J.J., Chen Y., Moore I., Lee J., Yuan M., Li Z.W., Karin M., Perret P. (2001). Prevention of fat-induced insulin resistance by salicylate. J. Clin. Investig..

[138] Zhu Y., Tchkonia T., Pirtskhalava T., Gower A.C., Ding H., Giorgadze N., Palmer A.K., Ikeno Y., Hubbard G.B., Lenburg M. (2015). The Achilles’ heel of senescent cells: From transcriptome to senolytic drugs. Aging Cell.

[139] Cheng T., Rodrigues N., Shen H., Yang Y., Dombkowski D., Sykes M., Scadden D.T. (2000). Hematopoietic stem cell quiescence maintained by p21cip1/waf1. Science.

[140] Muñoz-Espín D., Cañamero M., Maraver A., Gómez-López G., Contreras J., Murillo-Cuesta S., Rodríguez-Baeza A., Varela-Nieto I., Ruberte J., Collado M. (2013). Programmed cell senescence during mammalian embryonic development. Cell.

[141] Tinkum K.L., White L.S., Marpegan L., Herzog E., Piwnica-Worms D., Piwnica-Worms H. (2013). Forkhead box O1 (FOXO1) protein, but not p53, contributes to robust induction of p21 expression in fasted mice. J. Biol. Chem..

[142] Lopez-Guadamillas E., Fernandez-Marcos P.J., Pantoja C., Muñoz-Martin M., Martínez D., Gómez-López G., Campos-Olivas R., Valverde A.M., Serrano M. (2016). p21Cip1 plays a critical role in the physiological adaptation to fasting through activation of PPARα. Sci. Rep..

[143] Nemoto S., Matsumoto T., Taguchi K., Kobayashi T. (2014). Relationships among protein tyrosine phosphatase 1B, angiotensin II, and insulin-mediated aortic responses in type 2 diabetic Goto-Kakizaki rats. Atherosclerosis.

[144] Xi G., Shen X., Wai C., White M.F., Clemmons D.R. (2019). Hyperglycemia induces vascular smooth muscle cell dedifferentiation by suppressing insulin receptor substrate-1-mediated p53/KLF4 complex stabilization. J. Biol. Chem..

[145] Warburg O. (1956). On the origin of cancer cells. Science.

[146] Bensaad K., Tsuruta A., Selak M.A., Vidal M.N., Nakano K., Bartrons R., Gottlieb E., Vousden K.H. (2006). TIGAR, a p53-inducible regulator of glycolysis and apoptosis. Cell.

[147] Zhang C., Liu J., Wu R., Liang Y., Lin M., Liu J., Chan C.S., Hu W., Feng Z. (2014). Tumor suppressor p53 negatively regulates glycolysis stimulated by hypoxia through its target RRAD. Oncotarget.

[148] Vousden K.H., Ryan K.M. (2009). p53 and metabolism. Nat. Rev. Cancer.

[149] Cairns R.A., Harris I.S., Mak T.W. (2011). Regulation of cancer cell metabolism. Nat. Rev. Cancer.

[150] Schwartzenberg-Bar-Yoseph F., Armoni M., Karnieli E. (2004). The Tumor Suppressor p53 Down-Regulates Glucose Transporters GLUT1 and GLUT4 Gene Expression. Cancer Res..

[151] Jiang P., Du W., Wang X., Mancuso A., Gao X., Wu M., Yang X. (2011). p53 regulates biosynthesis through direct inactivation of glucose-6-phosphate dehydrogenase. Nat. Cell Biol..

[152] Chu K., Gao G., Yang X., Ren S., Li Y., Wu H., Huang Y., Zhou C. (2016). miR-512-5p induces apoptosis and inhibits glycolysis by targeting p21 in non-small cell lung cancer cells. Int. J. Oncol..

[153] Jin X., Kuang Y., Li L., Li H., Zhao T., He Y., Di C., Kang J., Yuan L., Yu B. (2022). A positive feedback circuit comprising p21 and HIF-1α aggravates hypoxia-induced radioresistance of glioblastoma by promoting Glut1/LDHA-mediated glycolysis. FASEB J..

[154] Chen H., Han C., Liu D., Wang F., Ha C. (2022). CBX3 promotes ovarian cancer progression by regulating p53/p21-mediated glucose metabolism via inhibiting NCOR2. Arch. Med. Sci..

[155] Mihaylova M.M., Shaw R.J. (2011). The AMPK signalling pathway coordinates cell growth, autophagy and metabolism. Nat. Cell Biol..

[156] Molnar Z., Millward A.B., Tse W., Demaine A.G. (2014). p21(WAF1/CIP1) Expression is Differentially Regulated by Metformin and Rapamycin. Int. J. Chronic Dis..

[157] Davies H., Bignell G.R., Cox C., Stephens P., Edkins S., Clegg S., Teague J., Woffendin H., Garnett M.J., Bottomley W. (2002). Mutations of the BRAF gene in human cancer. Nature.

[158] Nicholson R.I., Gee J.M., Harper M.E. (2001). EGFR and cancer prognosis. Eur. J. Cancer.

[159] Bos J.L. (1989). ras oncogenes in human cancer: A review. Cancer Res..

[160] Serrano M., Lin A.W., McCurrach M.E., Beach D., Lowe S.W. (1997). Oncogenic ras provokes premature cell senescence associated with accumulation of p53 and p16INK4a. Cell.

[161] Ridley A.J., Paterson H.F., Noble M., Land H. (1988). Ras-mediated cell cycle arrest is altered by nuclear oncogenes to induce Schwann cell transformation. EMBO J..

[162] Kohl N.E., Ruley H.E. (1987). Role of c-myc in the transformation of REF52 cells by viral and cellular oncogenes. Oncogene.

[163] Manu K.A., Chai T.F., Teh J.T., Zhu W.L., Casey P.J., Wang M. (2017). Inhibition of Isoprenylcysteine Carboxylmethyltransferase Induces Cell-Cycle Arrest and Apoptosis through p21 and p21-Regulated BNIP3 Induction in Pancreatic Cancer. Mol. Cancer Ther..

[164] Olson M.F., Paterson H.F., Marshall C.J. (1998). Signals from Ras and Rho GTPases interact to regulate expression of p21Waf1/Cip1. Nature.

[165] Bergo M.O., Gavino B.J., Hong C., Beigneux A.P., McMahon M., Casey P.J., Young S.G. (2004). Inactivation of Icmt inhibits transformation by oncogenic K-Ras and B-Raf. J. Clin. Investig..

[166] Ramanujulu P.M., Yang T., Yap S.Q., Wong F.C., Casey P.J., Wang M., Go M.L. (2013). Functionalized indoleamines as potent, drug-like inhibitors of isoprenylcysteine carboxyl methyltransferase (Icmt). Eur. J. Med. Chem..

[167] Winter-Vann A.M., Baron R.A., Wong W., dela Cruz J., York J.D., Gooden D.M., Bergo M.O., Young S.G., Toone E.J., Casey P.J. (2005). A small-molecule inhibitor of isoprenylcysteine carboxyl methyltransferase with antitumor activity in cancer cells. Proc. Natl. Acad. Sci. USA.

[168] Lau H.Y., Ramanujulu P.M., Guo D., Yang T., Wirawan M., Casey P.J., Go M.L., Wang M. (2014). An improved isoprenylcysteine carboxylmethyltransferase inhibitor induces cancer cell death and attenuates tumor growth in vivo. Cancer Biol. Ther..

[169] Teh J.T., Zhu W.L., Ilkayeva O.R., Li Y., Gooding J., Casey P.J., Summers S.A., Newgard C.B., Wang M. (2015). Isoprenylcysteine carboxylmethyltransferase regulates mitochondrial respiration and cancer cell metabolism. Oncogene.

[170] Wang M., Hossain M.S., Tan W., Coolman B., Zhou J., Liu S., Casey P.J. (2010). Inhibition of isoprenylcysteine carboxylmethyltransferase induces autophagic-dependent apoptosis and impairs tumor growth. Oncogene.

[171] Wang M., Tan W., Zhou J., Leow J., Go M., Lee H.S., Casey P.J. (2008). A small molecule inhibitor of isoprenylcysteine carboxymethyltransferase induces autophagic cell death in PC3 prostate cancer cells. J. Biol. Chem..

[172] Ding Y., Yang G., Wu Q. (2021). Autophagic dysfunction of β cell dysfunction in type 2 diabetes, a double-edged sword. Genes Dis..

[173] Collier J.J., Suomi F., Oláhová M., McWilliams T.G., Taylor R.W. (2021). Emerging roles of ATG7 in human health and disease. EMBO Mol. Med..

[174] Maheshwari M., Yadav N., Hasanain M., Pandey P., Sahai R., Choyal K., Singh A., Nengroo M.A., Saini K.K., Kumar D. (2022). Inhibition of p21 activates Akt kinase to trigger ROS-induced autophagy and impacts on tumor growth rate. Cell Death Dis..

[175] Luo Y., Zou P., Zou J., Wang J., Zhou D., Liu L. (2011). Autophagy regulates ROS-induced cellular senescence via p21 in a p38 MAPKα dependent manner. Exp. Gerontol..

[176] Brownlee M. (2005). The pathobiology of diabetic complications: A unifying mechanism. Diabetes.

[177] el-Deiry W.S., Tokino T., Velculescu V.E., Levy D.B., Parsons R., Trent J.M., Lin D., Mercer W.E., Kinzler K.W., Vogelstein B. (1993). WAF1, a potential mediator of p53 tumor suppression. Cell.

[178] Chen Z., Trotman L.C., Shaffer D., Lin H.K., Dotan Z.A., Niki M., Koutcher J.A., Scher H.I., Ludwig T., Gerald W. (2005). Crucial role of p53-dependent cellular senescence in suppression of Pten-deficient tumorigenesis. Nature.

[179] Yahagi N., Shimano H., Matsuzaka T., Najima Y., Sekiya M., Nakagawa Y., Ide T., Tomita S., Okazaki H., Tamura Y. (2003). p53 Activation in adipocytes of obese mice. J. Biol. Chem..

[180] Tanaka T., Suh K.S., Lo A.M., De Luca L.M. (2007). p21WAF1/CIP1 is a common transcriptional target of retinoid receptors: Pleiotropic regulatory mechanism through retinoic acid receptor (RAR)/retinoid X receptor (RXR) heterodimer and RXR/RXR homodimer. J. Biol. Chem..

[181] Nakatsuka A., Wada J., Hida K., Hida A., Eguchi J., Teshigawara S., Murakami K., Kanzaki M., Inoue K., Terami T. (2012). RXR antagonism induces G0 /G1 cell cycle arrest and ameliorates obesity by up-regulating the p53-p21(Cip1) pathway in adipocytes. J. Pathol..

[182] Cmielová J., Havelek R., Jiroutová A., Kohlerová R., Seifrtová M., Muthná D., Vávrová J., Rezáčová M. (2011). DNA damage caused by ionizing radiation in embryonic diploid fibroblasts WI-38 induces both apoptosis and senescence. Physiol. Res..

[183] Lehmann B.D., McCubrey J.A., Jefferson H.S., Paine M.S., Chappell W.H., Terrian D.M. (2007). A dominant role for p53-dependent cellular senescence in radiosensitization of human prostate cancer cells. Cell Cycle.

[184] Wang Y., Blandino G., Givol D. (1999). Induced p21waf expression in H1299 cell line promotes cell senescence and protects against cytotoxic effect of radiation and doxorubicin. Oncogene.

[185] Johnson-Arbor K., Dubey R. (2023). Doxorubicin. StatPearls.

[186] Sliwinska M.A., Mosieniak G., Wolanin K., Babik A., Piwocka K., Magalska A., Szczepanowska J., Fronk J., Sikora E. (2009). Induction of senescence with doxorubicin leads to increased genomic instability of HCT116 cells. Mech. Ageing Dev..

[187] Song Y.S., Lee B.Y., Hwang E.S. (2005). Dinstinct ROS and biochemical profiles in cells undergoing DNA damage-induced senescence and apoptosis. Mech. Ageing Dev..

[188] Zhang W.W., Fang X., Mazur W., French B.A., Georges R.N., Roth J.A. (1994). High-efficiency gene transfer and high-level expression of wild-type p53 in human lung cancer cells mediated by recombinant adenovirus. Cancer Gene Ther..

[189] Sturmlechner I., Zhang C., Sine C.C., van Deursen E.J., Jeganathan K.B., Hamada N., Grasic J., Friedman D., Stutchman J.T., Can I. (2021). p21 produces a bioactive secretome that places stressed cells under immunosurveillance. Science.

[190] Ahmad I.M., Abdalla M.Y., Aykin-Burns N., Simons A.L., Oberley L.W., Domann F.E., Spitz D.R. (2008). 2-Deoxyglucose combined with wild-type p53 overexpression enhances cytotoxicity in human prostate cancer cells via oxidative stress. Free Radic Biol. Med..

[191] Shatrov V.A., Ameyar M., Bouquet C., Cai Z., Stancou R., Haddada H., Chouaib S. (2000). Adenovirus-mediated wild-type-p53-gene expression sensitizes TNF-resistant tumor cells to TNF-induced cytotoxicity by altering the cellular redox state. Int. J. Cancer.

[192] Inoue T., Kato K., Kato H., Asanoma K., Kuboyama A., Ueoka Y., Yamaguchi S., Ohgami T., Wake N. (2009). Level of reactive oxygen species induced by p21Waf1/CIP1 is critical for the determination of cell fate. Cancer Sci..

[193] Zhang W.W., Li L., Li D., Liu J., Li X., Li W., Xu X., Zhang M.J., Chandler L.A., Lin H. (2018). The First Approved Gene Therapy Product for Cancer Ad-p53 (Gendicine): 12 Years in the Clinic. Hum. Gene Ther..

[194] Guo S.S., Zeller C., Chumlea W.C., Siervogel R.M. (1999). Aging, body composition, and lifestyle: The Fels Longitudinal Study. Am. J. Clin. Nutr..

[195] Tchkonia T., Morbeck D.E., Von Zglinicki T., Van Deursen J., Lustgarten J., Scrable H., Khosla S., Jensen M.D., Kirkland J.L. (2010). Fat tissue, aging, and cellular senescence. Aging Cell.

[196] Baker D.J., Weaver R.L., van Deursen J.M. (2013). p21 both attenuates and drives senescence and aging in BubR1 progeroid mice. Cell Rep..

[197] Baker D.J., Perez-Terzic C., Jin F., Pitel K.S., Niederländer N.J., Jeganathan K., Yamada S., Reyes S., Rowe L., Hiddinga H. (2008). Opposing roles for p16Ink4a and p19Arf in senescence and ageing caused by BubR1 insufficiency. Nat. Cell Biol..

[198] Montero J.C., Seoane S., Ocaña A., Pandiella A. (2011). Inhibition of SRC family kinases and receptor tyrosine kinases by dasatinib: Possible combinations in solid tumors. Clin. Cancer Res..

[199] Olave N.C., Grenett M.H., Cadeiras M., Grenett H.E., Higgins P.J. (2010). Upstream stimulatory factor-2 mediates quercetin-induced suppression of PAI-1 gene expression in human endothelial cells. J. Cell. Biochem..

[200] Peng S., Sen B., Mazumdar T., Byers L.A., Diao L., Wang J., Tong P., Giri U., Heymach J.V., Kadara H.N. (2016). Dasatinib induces DNA damage and activates DNA repair pathways leading to senescence in non-small cell lung cancer cell lines with kinase-inactivating BRAF mutations. Oncotarget.

[201] Ranelletti F.O., Maggiano N., Serra F.G., Ricci R., Larocca L.M., Lanza P., Scambia G., Fattorossi A., Capelli A., Piantelli M. (2000). Quercetin inhibits p21-RAS expression in human colon cancer cell lines and in primary colorectal tumors. Int. J. Cancer.

[202] Breccia M., Molica M., Alimena G. (2014). How tyrosine kinase inhibitors impair metabolism and endocrine system function: A systematic updated review. Leuk. Res..

[203] Seong H.A., Ha H. (2019). Thr55 phosphorylation of p21 by MPK38/MELK ameliorates defects in glucose, lipid, and energy metabolism in diet-induced obese mice. Cell Death Dis..

